# Glucose depletion enables *Candida albicans* mating independently of the epigenetic white-opaque switch

**DOI:** 10.1038/s41467-023-37755-8

**Published:** 2023-04-12

**Authors:** Guobo Guan, Li Tao, Chao Li, Ming Xu, Ling Liu, Richard J. Bennett, Guanghua Huang

**Affiliations:** 1 Department of Infectious Diseases, Shanghai Key Laboratory of Infectious Diseases and Biosafety Emergency Response, National Medical Center for Infectious Diseases, Huashan Hospital, Shanghai Institute of Infectious Disease and Biosecurity and State Key Laboratory of Genetic Engineering, School of Life Sciences, Fudan University, Shanghai, 200438 China; 2 State Key Laboratory of Mycology, Institute of Microbiology, Chinese Academy of Sciences, Beijing, 100101 China; 3 Molecular Microbiology and Immunology Department, Brown University, Providence, RI 02912 USA; 4 Shanghai Engineering Research Center of Industrial Microorganisms, Shanghai, 200438 China; 5 Shanghai Huashen Institute of Microbes and Infections, Shanghai, 200052 China

**Keywords:** Cellular microbiology, Fungal biology, Pathogens

## Abstract

The human fungal pathogen *Candida albicans* can switch stochastically and heritably between a “white” phase and an “opaque” phase. Opaque cells are the mating-competent form of the species, whereas white cells are thought to be essentially “sterile”. Here, we report that glucose depletion, a common nutrient stress, enables *C. albicans* white cells to undergo efficient sexual mating. The relative expression levels of pheromone-sensing and mating-associated genes (including *STE2/3*, *MFA1*, *MFα1*, *FIG1*, *FUS1*, and *CEK1/2*) are increased under glucose depletion conditions, while expression of mating repressors *TEC1* and *DIG1* is decreased. Cph1 and Tec1, factors that act downstream of the pheromone MAPK pathway, play opposite roles in regulating white cell mating as *TEC1* deletion or *CPH1* overexpression promotes white cell mating. Moreover, inactivation of the Cph1 repressor Dig1 increases white cell mating ~4000 fold in glucose-depleted medium relative to that in the presence of glucose. Our findings reveal that the white-to-opaque epigenetic switch may not be a prerequisite for sexual mating in *C. albicans* in nature.

## Abstract (web summary)

The pathogenic fungus *Candida albicans* can switch between a mating-competent form (“opaque” cells) and a form that is thought to be essentially sterile (“white” cells). Here, the authors show that glucose depletion, a common nutrient stress, enables *C. albicans* white cells to undergo efficient sexual mating.

## Introduction

**Fig. 1:**
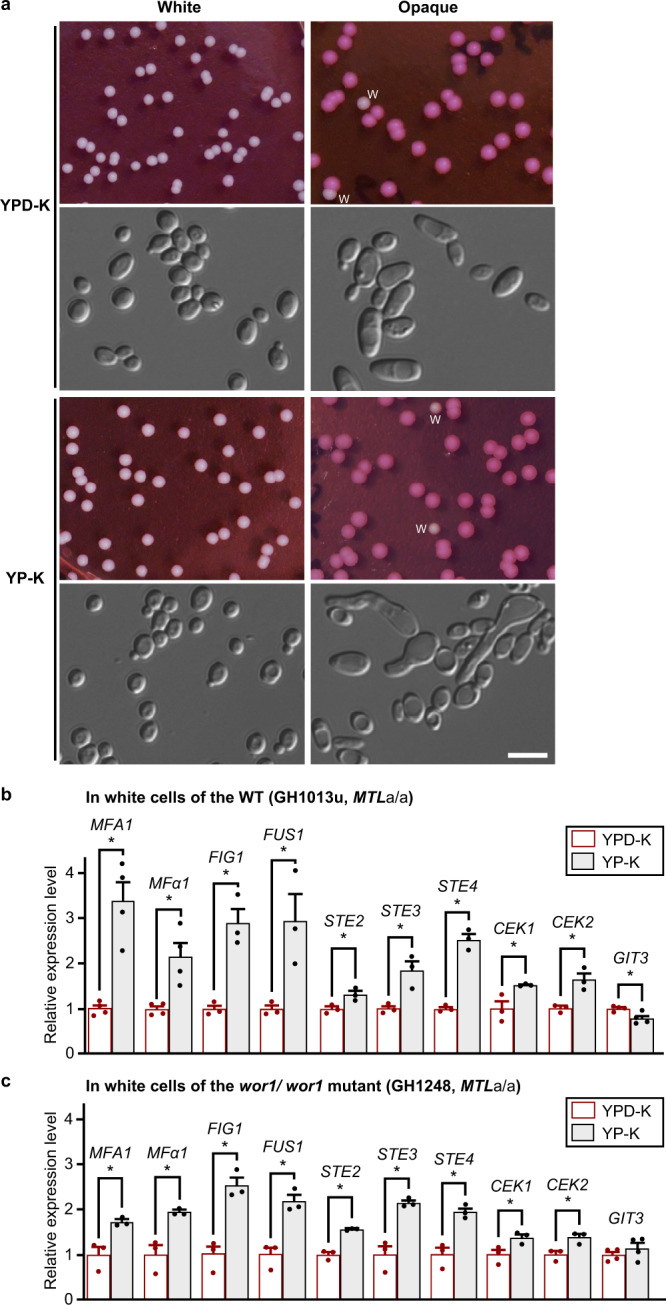
Glucose depletion induces the expression of mating-associated genes in *C. albicans* white cells. **a** Colony and cellular morphologies of white and opaque phenotypes. White or opaque cells of strain GH1013u (WT, *MTL***a**/**a**) were plated and grown on YPD-K (for 3 days) or YP-K (for 5 days) medium supplemented with the red dye phloxine B at 25 °C. w, white colony. Scale bar, 10 μm. One representative image of three independent experiments is shown. Relative expression levels of mating-associated genes in white cells of the WT strain GH1013u (**b**) and *wor1/wor1* mutant (GH1248, **c**). *C. albicans* cells were initially grown overnight in SCD liquid medium. Approximately 1 × 10^7^ of cells were spotted on YP-K (w/o glucose) and YPD-K (with 2% glucose) media and incubated at 25 °C for 3 days, respectively. Gene *GIT3* served as a control (its expression was not increased in response to glucose depletion). The relative expression level of each gene in YPD-K medium was set as ‘1’. WT, wild type. Data are presented as the average ± SEM. Statistical analyses for (**b** and **c**): YP-K vs. YPD-K, two-sided *t* test, *n*  =  3–4. **b**: *MFA1*, *P*  =  0.0015; *MFα1*, *P*  =  0.0099; *FIG1*, *P*  =  0.0050; *FUS1*, *P*  =  0.0333; *STE2*, *P*  =  0.0313; *STE3, P*  =  0.0174; *STE4*, *P*  =  0.0003; *CEK1*, *P*  =  0.0403; *CEK2*, *P*  =  0.0144; *GIT3*, *P*  =  0.0192; (**c**): *MFA1*, *P*  =  0.0197; *MFα1*, *P*  =  0.0110; *FIG1*, *P*  =  0.0043; *FUS1*, *P*  =  0.0061; *STE2*, *P*  =  0.0010; *STE3, P*  =  0.0045; *STE4*, *P*  =  0.0064; *CEK1*, *P*  =  0.0487; *CEK2*, *P*  =  0.0250; *GIT3*, *P*  =  0.3337. Source data are provided as a Source Data file.

Sexual reproduction is ubiquitous across eukaryotic organisms. In fungi, sexual reproduction drives the evolution of new traits and generates diversity that can enable adaptation to environmental changes^1–3^. Although certain aspects of sexual reproduction are conserved in fungi, such as pheromone signaling via a MAPK transduction pathway, there are also important differences in the regulation of this program between different species^1^. A unique regulatory mechanism is observed in the human pathogenic fungus *Candida albicans* and its closely related species *Candida tropicalis* and *Candida dubliniensis*, where sexual mating is regulated by an epigenetic switch^4–7^. In each of these species, cells must switch from the “sterile” white state to the mating-competent opaque state to undergo efficient sexual mating. Besides mating competence, *C. albicans* white and opaque cells differ in a number of phenotypic aspects including colony and cellular morphologies (Fig. 1a) and virulence in different infection models^4,8,9^.

The white-opaque phenotypic switch occurs ectopically at low frequency under standard culture conditions but can be induced by a variety of environmental cues such as high levels of CO_2_ or N-acetylglucosamine (GLcNAc), as well as in response to changes in pH or temperature^8^. This phenotypic transition is operated by a transcriptional circuit centered on the key regulators Wor1 and Efg1 in *C. albicans*^10–13^. Wor1 is a fungal-specific transcription factor that is considered the master regulator of *C. albicans* white-opaque switching and is essential for the opaque cell type^10–12^.

*C. albicans* opaque cells mate approximately one million times more efficiently than white cells^4^. It has long been thought, therefore, that switching from the white to the opaque state is essentially a prerequisite for mating. However, several studies have indicated that white cells are able to respond to pheromone signals and may play a critical role in promoting sexual reproduction^14,15^. When mixed with opaque cells or exposed to pheromone, *C. albicans* white cells exhibited increased expression levels of a subset of mating-associated genes and formed “sexual biofilms” that facilitated opaque cell mating^16^. In a related study, we showed that *C. albicans* white cells secrete pheromone and promote opaque cell mating in a mixed culture system containing both cell types^15^. These studies indicate that although white cells are unable to mate efficiently under standard culture conditions, the major components of the mating response MAPK signaling pathway can be activated and can generate a conducive environment for opaque cell mating.

Although *C. albicans* white cells can exhibit mating-related responses under certain conditions, cell–cell conjugation between white cells is limited due to the low expression of pheromone MAPK signaling genes such as *STE4*, *CST5*, and *CEK1*/*CEK2* (encoding the β subunit of the G-protein complex, the MAPK scaffold, and the terminal MAP kinases, respectively)^17^. Indeed, ectopic expression of *STE4*, *CST5*, and *CEK2*, both individually and in combination, increases mating between white cells, demonstrating that the white-opaque switch controls mating through regulation of the pheromone MAPK signaling pathway^17^.

Nutrient limitation is a common stress encountered by *C. albicans* cells in its natural niches, and we recently reported that glucose depletion activates the pheromone signaling MAPK pathway and promotes efficient same-sex (homothallic) mating by *C. albicans* opaque cells^18^. The Hsf1/Hsp90-mediated stress response pathway plays a central role in this regulation^18^. These findings suggested that it may be possible to alleviate bottlenecks in the expression of mating-associated genes and enable sexual mating in white cells through similar perturbations in nutrients.

In this study, we demonstrate that depletion of glucose activates the pheromone MAPK pathway in *C. albicans* white cells, and thereby promotes their ability to undergo sexual mating. The MAPK Cek2 and transcription factor Cph1 function as positive regulators of white cell mating, whereas the transcription factors Tec1 and Dig1 inhibit white cell mating. Given glucose limitation is a common stress encountered by *C. albicans* in its mammalian host, our findings indicate that the white-opaque switch may not be essential for efficient mating in nature. Thus, this epigenetic switch, which is a relatively newly evolved regulatory mechanism in *C. albicans* and closely associated species, can be bypassed under certain environmental conditions.

## Results

### Glucose depletion induces the expression of mating-associated genes in *C. albicans* white cells

Most media used for culturing *C. albicans* contains 1–2% of glucose (w/v), which is much higher than the level of glucose in environmental niches (e.g., the lower animal gut, mucosal surfaces, and soil environments)^19,20^. Glucose depletion has multiple effects on the life cycle of microbes^18,21,22^. We previously found that glucose depletion induces the expression of mating-associated genes and promotes same-sex mating in opaque cells of *C. albicans*^18^. To test the effect of glucose depletion on white cells, we examined the relative expression levels of mating-associated genes in *C. albicans* that are homozygous for the *MTL***a** mating type locus. Media contained either no glucose (YP-K) or 2% glucose (YPD-K) in these tests. As shown in Fig. 1b, the relative expression levels of several pheromone MAPK genes were significantly increased in white cells grown in YP-K medium compared to those grown in YPD-K medium. These genes included *MFA1*, *MFα1*, *STE2*, and *STE3* encoding the **a**- and α-pheromones and their receptors, respectively, *CEK1* and *CEK2* encoding the terminal MAPKs of the pheromone signaling pathway, and *STE4* encoding the ortholog of the *S. cerevisiae* G protein β-subunit Ste4^17,23^. Two additional genes, *FIG1* and *FUS1*^17,23–26^, were also transcriptionally upregulated in the YP-K medium relative to the YPD-K medium. Both of these genes are integral to mating in *S. cerevisiae* and Fig. 1 protein is localized to mating projections in *C. albicans*^26,27^.

Since white cells can switch to the opaque phase at low frequency which would increase the expression of mating-associated genes, we also performed qRT-PCR assays in a white-locked *wor1/wor1* mutant. A similar expression pattern was observed in the *wor1/wor1* mutant to that of the WT strain, with higher expression of the set of mating-related genes in YP-K medium than in YPD-K medium (Fig. 1c). In the YP-K and YPD-K media, K_2_HPO_4_ was used as a pH buffer. These results reveal that glucose depletion induces the expression of mating-associated genes in *C. albicans* white cells.

### Effect of different culture media on white cell mating

**Fig. 2:**
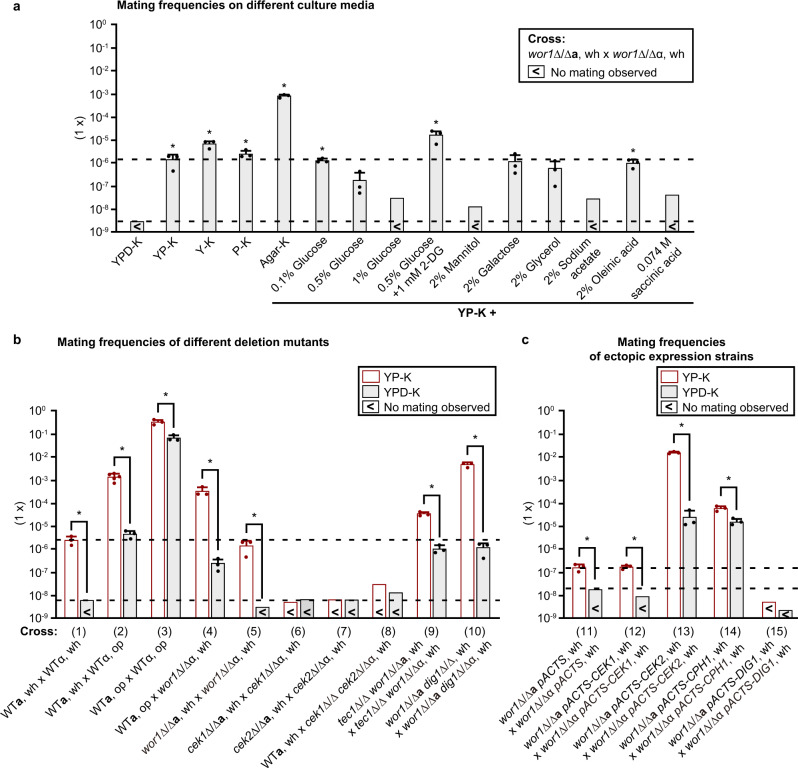
Mating frequencies of the WT and mutated strains of *C. albicans*. **a** Mating frequencies of the *wor1/wor1***a** x *wor1/wor1*α cross on different media. Strains used: *wor1/wor1***a**, GH1248; *wor1/wor1*α, GBS2347. **b** and **c** Mating frequencies of the WT, mutated, or ectopic expression strains on YP-K and YPD-K media. The mixture of “**a**” and α mating partners (~3 × 10^7^ cells for each strain) was spotted and cultured on YP-K and YPD-K media at 25 °C for 7 days. This figure is associated with Table 1 and S1. Detailed methods and strain information are described in the methods section and supplementary materials (Tables S2 and S3). *n* = 3–5 independent experiments and the results represent the average ± SD. “<” indicates no mating progeny colonies observed; wh, white cells; op, opaque cells. Statistical differences were determined by two-sided unpaired Student’s *t*-test, * = *P* < 0.05. In (**a**), compared to YPD-K, *P* values for quantitative mating assays on different media are: YP-K, *P* = 0.0437; Y-K, *P* = 0.0077; P-K, *P* = 0.0098; agar-K, *P* = 0.0005; 0.1% glucose, *P* = 0.0010; 0.5% glucose, *P* = 0.1886; 0.5% glucose + 1 mM 2-DG, *P* = 0.0353; 2% galactose, *P* = 0.1464; 2% glycerol, *P* = 0.1372; 2% oleinic acid, *P* = 0.0136. In (**b**), YP-K vs. YPD-K: cross (1), *P* = 0.0163; cross (2), *P* = 0.0035; cross (3), *P* = 0.0057; cross (4), *P* = 0.0167; cross (5), *P* = 0.0437; cross (9), *P* = 0.0019; cross (10), *P* = 0.0020. In (**c**), YP-K vs. YPD-K: cross (11), *P* = 0.0298; cross (12), *P* = 0.0013; cross (13), *P* < 0.0001; cross (14), *P* = 0.0097. Source data are provided as a Source Data file.

To further investigate the effect of nutrient states on *C. albicans* white cell mating, we performed quantitative mating assays with the white-locked strains GH1248 and GBS2347 on several culture media (Table S1 and Fig. 2a). First, we found that there was a dosage effect of glucose on white cell mating. The presence of 0.5% of glucose dramatically decreased the mating efficiency and 1.0% of glucose completely blocked mating. Second, compared to that on YP-K medium, the mating efficiency was slightly increased on Y-K (10 g/L yeast extract, 2.5 g/L K_2_HPO_4_, 20 g/L agar) and P-K (20 g/L peptone, 2.5 g/L K_2_HPO_4_, 20 g/L agar) media. However, it was increased 571 times on agar medium (2.5 g/L K_2_HPO_4_, 20 g/L agar), which represents an extremely poor nutrient condition.

To examine whether altered glucose utilization leads to mating competency, we performed mating assays using white cells of the *gpr1/gpr1* and *hgt12/hgt12* mutants, we found that deletion of these two genes caused a slight increase of mating competency on both YP-K and YPD-K media (Table S2, crosses 32 and 33). This limited effect on mating could be due to the fact that there are multiple pathways involved in the regulation of glucose and nutrient utilization and there are multiple glucose transporters in *C. albicans*. These pathways and transporters play redundant roles in glucose utilization. To further examine the effect of altered glycolysis, we added the competitive inhibitor of glycolysis, 2-deoxy-D-glucose (2-DG), to the medium and found that the presence of low levels of 2-DG promoted white cell mating (Table S1, crosses 7 and 9). These results suggest that depletion of glucose plays a major role in the induction of white cell mating and that yeast extract and peptone have an additive effect.

We also examined the impact of different carbon sources on white cell mating (Table S1, crosses 10 to 15). Oleinic acid (added to YP-K medium) had no significant effect on mating; galactose and glycerol reduced the mating efficiency; and mannitol, sodium acetate, or succinic acid completely blocked white cell mating. These results were reasonable because *C. albicans* can utilize mannitol but not glycerol and oleinic acid efficiently as a carbon source in the absence of glucose. Sodium acetate and succinic acid could affect medium pH after a period of cell growth and thus cause cellular stress to *C. albicans*.

### Glucose depletion sensitizes the pheromone response and promotes the development of mating projections in white cells

**Fig. 3:**
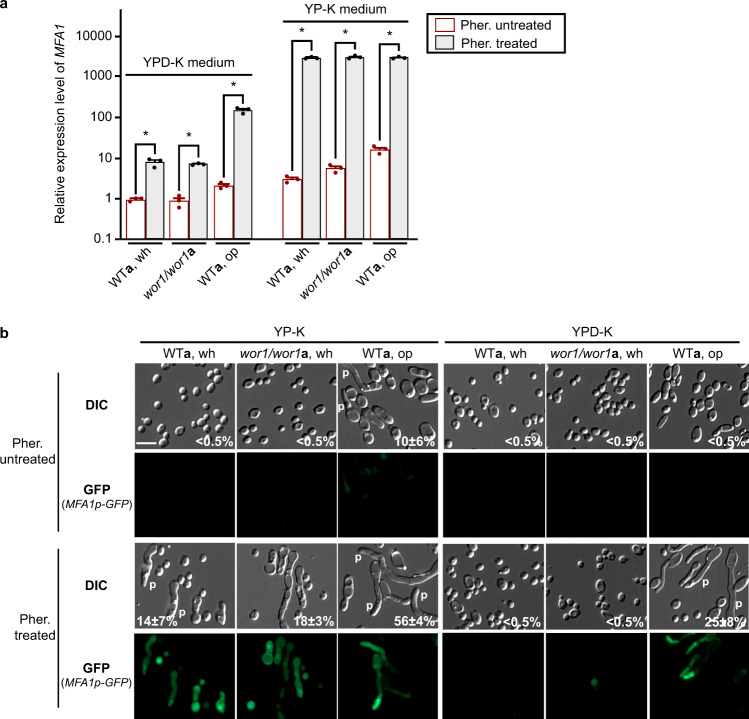
Glucose depletion sensitizes the pheromone response pathway and promotes the development of mating projection in *C. albicans* white cells. Strains used: WT**a**, GH1013u (*MTL****a****/****a***); *wor1/wor1***a**, (GH1248, *MTL****a****/****a***). Approximately 1 × 10^7^ of white or opaque cells were spotted onto YP-K or YPD-K media and incubated at 25 °C for 3 days. Three μL of 200 μM α-pheromone was added onto the spot every 24 h (at 0, 24, and 48 h). After 3 days of incubation, *C. albicans* cells were collected for qRT-PCR or microscopy assays. **a** Relative expression levels of *MFA1* in the WT white cells (two-sided *t* test, *n*  =  3, YPD-K, *P*  =  0.0027; YP-K, *P*   = 5.4E-06) and opaque cells (two-sided *t* test, *n*  =  3, YPD-K, *P*  =  0.0004; YP-K, *P*  = 5.5E-06) and white-locked *wor1/wor1* cells (two-sided *t* test, *n*  =  3, YPD-K, *P*  =  2.6E-05; YP-K, *P*  = 4.9E-05) when treated with or without α-pheromone in YP-K and YPD-K media. The relative expression level of *MFA1* in WT white cells (untreated with α-pheromone) in YPD-K medium was set as ‘1’. Data are presented as the average ± SEM. *P < 0.05 (Student’s *t* test, two-sided). **b** Development of mating projections in the WT (white and opaque) and white-locked *wor1/wor1* cells when treated with or without α-pheromone in YP-K and YPD-K media. The GFP reporter was introduced into the strains GH1013u and GH1248 and was under the control of the *MFA1* promoter. Scale bar, 10 μm. One representative image of three independent experiments is shown. Source data are provided as a Source Data file.

We predicted that increased expression of mating-associated genes in glucose-depleted medium would promote the pheromone response in white cells. To test this hypothesis, we treated WT white and opaque *MTL***a** cells and white-locked *wor1/wor1 MTL***a** cells with α-pheromone. The expression of *MFA1* was used as a reporter of the pheromone response as this gene is not expressed in either white or opaque cells in the absence of α-pheromone^28^. As shown in Fig. 3a, pheromone treatment increased the expression levels of *MFA1* about tenfold in WT and *wor1/wor1* white cells on YPD-K medium. In contrast, *MFA1* expression levels increased more than 531 times in pheromone-treated white cells grown on YP-K medium, comparable to pheromone-induced levels in opaque cells. These findings indicate that glucose depletion sensitizes white cells to pheromone treatment.

To verify that white cells respond more strongly to pheromone under glucose depletion conditions, microscopy was performed using a set of reporter strains in which GFP was under the control of the *MFA1* promoter. Consistent with the qRT-PCR results, GFP signal was observed in white cells when treated with α-pheromone in YP-K medium but not in YPD-K medium (Fig. 3b). Under the same conditions, we also observed the development of mating projections in both WT and *wor1/wor1* white cells in response to pheromone (Fig. 3b). To further verify the cell identity, we examined the relative expression levels of mating-associated, white-specific, and opaque-specific gene expression using qRT-PCR assays. As shown in Fig. S1, the expression of white-specific genes was increased in white cells and the expression of mating-specific genes was increased in both opaque and white cells. Our findings reveal that glucose depletion promotes the mating response, including the development of mating projections, in *C. albicans* white cells.

To characterize protein levels in white cells, we performed a comparative analysis of the proteome in a white-locked *wor1/wor1* strain grown on YP-K and YPD-K media (Fig. S2 and Dataset S1). We found that the expression profile on YP-K medium was different from the expression profile of opaque cells^29,30^. The differentially expressed proteins included both phase-dependent and phase-independent ones. As expected, a large set of metabolism-associated genes were differentially expressed under the two culture conditions. These results indicate that *C. albicans* white cells grown on YP-K medium did not switch to the opaque phenotype. Taken together with the transcriptional expression profile, the proteomic results confirm that cells in the white state are responding to pheromone without undergoing switching.

### Glucose depletion promotes efficient mating in white cells

To test whether glucose depletion would facilitate sexual mating by white cells, we performed quantitative mating assays as reported in Fig. 2 and Tables 1 and S2. When both partners were white cells (cross 1), *C. albicans* mated at least >391 times more efficiently on YP-K medium than on the glucose-containing YPD-K medium (with no mating progeny obtained on YPD-K medium). When one mating partner was white and the other was opaque (crosses 2 and 16), *C. albicans* mated 298 and 163 times more efficiently on YP-K medium than on YPD-K medium. Although depletion of glucose had no notable effect on white-to-opaque switching^18^, to verify that glucose depletion promotes white cell mating, we also examined the mating competency of white-locked *wor1/wor1* cells (crosses 4, 5, and 17). As shown in Fig. 2 and Tables 1 and S2, when *WOR1* was deleted from both mating partners, *C. albicans* mated on YP-K medium at least >467 times more efficiently than on YPD-K medium (no mating obtained on YPD-K medium, Cross 5). When one mating partner was white *wor1/wor1* cells and the other was WT opaque cells (crosses 4 and 17), mating efficiencies increased 1375 and 324 times on YP-K medium relative to YPD-K medium. Moreover, mating between two opaque partners on YP-K medium was five times higher than that on YPD-K medium (cross 3). These results demonstrate that depletion of glucose has a remarkable effect in enabling *C. albicans* white cell mating but only a relatively small effect on increasing opaque cell mating.

**Table 1:** Mating frequencies of the WT and mutated strains of *C. albicans* on YP-K and YPD-K media

Cross		On YP-K	On YPD-K	Fold change (YP-K/YPD-K)
(1)	*WT****a****, wh* ***x*** *WTα, wh*	(2.5 ± 1.1) × 10^−6^	<6.4 × 10^−9*^	>391
(2)	*WT****a***, *wh* ***x*** *WTα, op*	(1.4 ± 0.5) × 10^−3*^	(4.7 ± 1.8) × 10^−6*^	298
(3)	*WT****a****, op ****x*** *WTα, op*	(3.3 ± 0.8) × 10^−1*^	(6.7 ± 2.1) × 10^−2*^	5
(4)	*WT****a****, op* ***x*** *wor1Δ/Δα, wh*	(3.3 ± 1.5) × 10^−4*^	(2.4 ± 1.4) × 10^−7*^	1,375
(5)	*wor1Δ/Δ****a****, wh* ***x*** *wor1Δ/Δα, wh*	(1.4 ± 0.9) × 10^−6^	<3.0 × 10^−9*^	>467
(6)	*cek1Δ/Δ****a****, wh ****x*** *cek1Δ/Δα, wh*	<5.1 × 10^−9*^	<6.9 × 10^−9^	NA
(7)	*cek2Δ/Δ****a****, wh* ***x*** *cek2Δ/Δα, wh*	<6.5 × 10^−9*^	<6.6 × 10^−9^	NA
(8)	*WT****a****, wh* ***x*** *cek1Δ/Δ cek2Δ/Δα, wh*	<3.0 × 10^−8*^	<1.3 × 10^−8^	NA
(9)	*tec1Δ/Δ wor1Δ/Δ****a****, wh* ***x*** *tec1Δ/Δ wor1Δ/Δα, wh*	(3.4 ± 0.8) × 10^−5*^	(1.0 ± 0.4) × 10^−6*^	34
(10)	*wor1Δ/Δ****a*** *dig1Δ/Δ, wh* ***x*** *wor1Δ/Δα dig1Δ/Δ,wh*	(4.7 ± 1.1) × 10^−3*^	(1.2 ± 0.7) × 10^−6*^	3,916
(11)	*wor1Δ/Δ****a*** *pACTS, wh* ***x*** *wor1Δ/Δα pACTS, wh*	(1.6 ± 0.9) × 10^−7^	<1.7 × 10^−8*^	>9
(12)	*wor1Δ/Δ****a*** *pACTS-CEK1, wh* ***x*** *wor1Δ/Δα pACTS-CEK1, wh*	(1.7 ± 0.3) × 10^−7^	<9 × 10^−9*^	>19
(13)	*wor1Δ/Δ****a*** *pACTS-CEK2, wh* ***x*** *wor1Δ/Δα pACTS-CEK2, wh*	(1.5 ± 0.1) × 10^−2*^	(2.5 ± 2.2) × 10^−5*^	600
(14)	*wor1Δ/Δ****a*** *pACTS-CPH1, wh* ***x*** *wor1Δ/Δα pACTS-CPH1, wh*	(5.8 ± 1.5) × 10^−5*^	(1.6 ± 0.5) × 10^−5*^	4
(15)	*wor1Δ/Δ****a*** *pACTS-DIG1, wh* ***x*** *wor1Δ/Δα pACTS-DIG1, wh*	<5.1 × 10^−9*^	<2.3 × 10^−9^	NA

This table is associated with Fig. 2b and c. The mixture of “**a**” and α mating partners (~3 × 10^7^ cells for each strain) was spotted and cultured on YP-K and YPD-K media at 25 °C for 7 days. The mating mixture was then replated onto SCD selection media to determine mating frequency. *n* = 3–5 independent experiments, and the results represent the average ± SD. Statistical differences were determined by two-sided unpaired Student’s *t*-test, * = *P* < 0.05. For YP-K vs. YPD-K comparisons: cross (1), *P* = 0.0163; cross (2), *P* = 0.0035; cross (3), *P* = 0.0057; cross (4), *P* = 0.0167; cross (5), *P* = 0.0437; cross (9), *P* = 0.0019; cross (10), *P* = 0.0020; cross (11), *P* = 0.0298; cross (12), *P* = 0.0013; cross (13), *p* < 0.0001; cross (14), *P* = 0.0097. For comparisons between different crosses under YP-K condition: cross (1) vs. cross (2), *P* = 0.0034; cross (1) vs. cross (3), *P* = 0.0022; cross (1) vs. cross (6), *P* = 0.0162; cross (1) vs. cross (7), *P* = 0.0163; cross (1) vs. cross (8), *P* = 0.0168; cross (5) vs. cross (4), *P* = 0.0169; cross (5) vs. cross (9), *P* = 0.0020; cross (5) vs. cross (10), *P* = 0.0020; cross (11) vs. cross (12), *P* = 0.9148; cross (11) vs. cross (13), *p* < 0.0001; cross (11) vs. cross (14), *P* = 0.0026; cross (11) vs. cross (15), *P* = 0.0082. For comparisons between different crosses under YPD-K condition: cross (1) vs. cross (2), *P* = 0.0110; cross (1) vs. cross (3), *P* = 0.0050; cross (5) vs. cross (4), *P* = 0.0435; cross (5) vs. cross (9), *P* = 0.0107; cross (5) vs. cross (10), *P* = 0.0403; cross (11) vs. cross (13), *P* = 0.1109; cross (11) vs. cross (14), *P* = 0.0065. “<” indicates no progeny colonies observed; wh, white cells; op, opaque cells. The strain information and detailed methods for each cross are presented in Tables S2 and S3. Source data are provided as a Source Data file.

### Roles of the pheromone MAPK signaling pathway and transcription factors Cph1, Tec1, and Dig1 in white cell mating

We next tested the role of the conserved pheromone MAPK signaling pathway in supporting *C. albicans* white cell mating. As expected, deletion of the α-pheromone receptor-encoding gene *STE2* blocked white cell mating (Table S2, cross 31). Deletion of either one or both of the *CEK1*/*2* MAPK genes significantly decreased the efficiency of white cell mating on YP-K medium (crosses 6, 7, 8, 29, and 30). Consistently, two previous reports also found that inactivation of *CEK1* or *CEK2* decreases the mating efficiency in *C. albicans*^23,31^. However, ectopic expression of *CEK2* but not *CEK1* in the white-locked *wor1/wor1* mutant highly enhanced mating on both YP-K and YPD-K media (cross 11, 12, and 13). Mating of the strain ectopically expressing *CEK2* was (1.5 ± 0.1) × 10^−2^ on YP-K medium, which was 600 times higher than that on YPD-K medium (cross 13). These results suggest that both Cek1 and Cek2 play roles in mating of WT cells on regular media, while white cell mating in glucose-depleted conditions was highly sensitive to Cek2 expression levels.

The pheromone MAPK signaling pathway activates the Cph1 transcription factor which is essential for *C. albicans* mating^23^. We found that ectopic expression of *CPH1* in white cells significantly increased mating on both YP-K and YPD-K media (crosses 11 and 14). The conserved TEA/ATTS transcription factor Tec1 is a target of the Cek2 kinase and involved in the regulation of the white cell pheromone response and development of “sexual biofilms” in *C. albicans*^32–34^. As shown in Fig. 2b and Table 1 (crosses 5 and 9), deletion of *TEC1* in the *wor1/wor1* mutant promoted white cell mating on both YP-K and YPD-K media. Ectopic expression of *CPH1* or deletion of *TEC1* increased mating frequencies ~941- or 333-fold, respectively, on YPD-K medium (crosses 5, 9, 11, and 14), suggesting that these genetic modifications at least partially override the repressing effect of glucose. Consistent with Tec1 negatively impacting mating, ectopic *TEC1* expression completely blocked white cell mating on both YP-K and YPD-K media (crosses 19, 20, 21, and 22) and reduced the efficiency of opaque cell mating (crosses 23 and 24). These findings indicate that Cph1 and Tec1 play critical, but opposing, roles in *C. albicans* white cell mating.

*C. albicans* Dig1 is the single ortholog of *S. cerevisiae* Dig1 and Dig2, which are MAPK-responsive inhibitors of Ste12, the *S. cerevisiae* ortholog of *C. albicans* Cph1^35^. Given the critical function of Dig1 in regulating *S. cerevisiae* Ste12 and *C. albicans* Cph1 expression^35,36^, we examined the role of Dig1 in *C. albicans* white cell mating. As shown in Fig. 2b and Table 1, deletion of *DIG1* in the *wor1/wor1* mutant increased mating 400 fold on YPD-K medium, whereas the efficiency was increased 3357 fold on YP-K medium compared to the control expressing *DIG1* (crosses 5 and 10). Of note, the mating efficiency of the *wor1Δ/Δ dig1Δ/Δ* double mutant (cross 10) on YP-K medium was ~4000 fold higher than that on YPD-K medium. Conversely, ectopic expression of *DIG1* blocked white cell mating on both YP-K and YPD-K media (crosses 11 vs. 15, and 21 vs. 26) and decreased the efficiency of opaque cell mating (crosses 19 vs. 25, and 23 vs. 27). These results establish that Dig1 functions as a key negative regulator of glucose depletion-induced white cell mating in *C. albicans*.

### Expression and roles of Cek2, Cph1, Tec1, and Dig1 in white cell mating under glucose depletion conditions

**Fig. 4:**
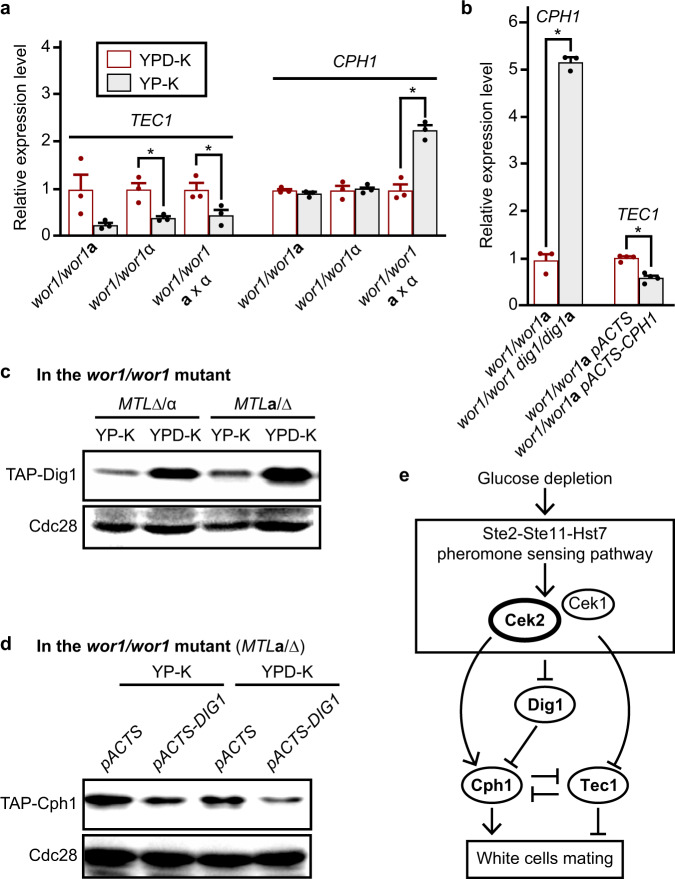
Relationships among the Cph1, Tec1, and Dig1 transcription factors. **a** Relative expression levels of *CPH1* (YP-K vs. YPD-K, two-sided *t* test, *n*  =  3-4, *wor1/wor1***a**, *P*  =  0.0767; *wor1/wor1*α, *P*  = 0.8611; *wor1/wor1* **a** x α, *P*  = 0.0020) and *TEC1* (two-sided *t* test, *n*  =  3, *wor1/wor1***a**, *P*  =  0.0834; *wor1/wor1*α, *P*  = 0.0155; *wor1/wor1* **a** x α, *P*  = 0.0400) in the *wor1/wor1* mutants. The relative expression level of each gene in YPD-K medium was set as “1”. Data are presented as the average ± SEM. **b** Relative expression levels of *CPH1* and *TEC1* in the *wor1/wor1*, *wor1/wor1 dig1/dig1* double mutants, *wor1/wor1***a** pACTS, and *wor1/wor1***a** pACTS-CPH1 strains in YP-K. The relative expression levels of the controls (the *wor1/wor1* and *wor1/wor1***a** pACTS mutants) were set as “1”. Data are presented as the average ± SEM. *P < 0.05 (Student’s *t*-test, two-sided). **c** Protein expression level of Dig1 in YP-K and YPD-K media. The TAP-tagged *DIG1* was introduced into *wor1/wor1* mutants (*MTL***a**/Δ and *MTL*Δ/α*)*. **d** Protein expression level of Cph1 in the control and *DIG1* overexpression strain. The TAP-tagged *CPH1* and pACTS-DIG1 were introduced in the *wor1/wor1* mutant (*MTL***a**/Δ). Cdc28 served as loading controls for Western blotting assays for (**c**) and (**d**). **e** Schematic model of regulatory pathway controlling white cell mating under glucose depletion conditions. Glucose depletion activates the pheromone MAPK signaling pathway and thus promotes white cell mating in *C. albicans*. The terminal MAPK Cek2 and transcription factor Cph1 play a positive role, whereas transcription factors Tec1 and Dig1 function as negative regulators of white cell mating. Source data are provided as a Source Data file.

To define the differential roles of these MAPK regulators in the presence and absence of glucose, we examined their RNA or protein levels in white cells (Figs. 1 and S3). Both the RNA and protein expression levels of *CEK2* were increased in YP-K medium compared to those in YPD-K medium (Figs. 1b, c, and S3), whereas the expression levels of Tec1 were decreased in YP-K medium (Fig. 4a and Fig. S3). The protein expression levels of Dig1 were decreased in both “**a**” and “α” cells in YP-K medium compared to that in YPD-K medium (Fig. 4c). As aforementioned, Cek2 plays a positive role, whereas Tec1 and Dig1 function as negative regulators in the control of white cell mating. Therefore, these expression differences were in line with their roles in promoting/inhibiting white cell mating in *C. albicans*.

The relative transcriptional expression levels of *CPH1* were comparable in YP-K and YPD-K media in the single strain cultures (“**a**” or “α”, Fig. 4a). However, the expression level of *CPH1* in YP-K medium was much higher than that in YPD-K medium in the “**a**” and “α” cell mixed cultures (Fig. 4a). Deletion of *DIG1* also increased the transcriptional expression of *CPH1* and, consistently, ectopic expression of *DIG1* reduced the expression level of Cph1 (Fig. 4b and d). Ectopic expression of *CPH1* repressed the expression of *TEC1* (Fig. 4b).

These results highlight the regulatory interplay between Cph1, Tec1, and Dig1 to control white cell mating under glucose depletion conditions. Based on the regulatory relationships found here and previous publications^17,32,33,36^, we propose that glucose depletion promotes white cell mating through the control of the expression of these key regulators and activation of the pheromone MAPK pathway in *C. albicans*. A model is shown for how Cek2, Cph1, Tec1, and Dig1 may coordinately regulate white cell mating (Fig. 4e).

## Discussion

There are advantages and disadvantages associated with undergoing sexual reproduction in eukaryotic organisms^37,38^. Although sexual reproduction creates genetic and phenotypic diversity enabling rapid adaptation to new environments, its costs are also significant (*e.g*., it takes additional energy, typically produces fewer offspring and can result in the loss of favored genetic configurations compared to asexual reproduction). In fungal species, an enormous diversity of regulatory mechanisms has evolved to control sexual reproduction^1,39^. In the pathobiont *C. albicans*, the white-opaque switch provides a stringent additional tier of control for sexual reproduction^4^, which may represent a balancing strategy between sexual and asexual life cycles. However, it is still unclear as to what benefits the white-opaque switch provides to *C. albicans*, or why mating gained this extra level of regulatory control in this species and two close relatives.

In this study, we demonstrate that glucose depletion alleviates the bottlenecks on sexual mating in *C. albicans* white cells due to activation of the pheromone MAPK pathway and downregulation of mating repressors such as Tec1 and Dig1 (Fig. 4e). These expression changes act in combination to elevate the mating competency of WT white cells by more than two orders of magnitude. Given that the white-opaque switch evolved relatively recently in *C. albicans* and closely related species, this mating strategy could represent a holdover from a more ancestral mode of sexual reproduction.

We previously reported that glucose depletion supports the development of mating projections and same-sex mating in opaque cells of *C. albicans* through the regulation of the pheromone signaling MAPK and stress-response Hsf1-Hsp90 signaling pathways^18^. In haploid cells of *S. cerevisiae*, depletion of glucose promotes an elongated morphology and invasive growth through activation of the Ras GTPase controlled cAMP/PKA signaling and Ste11-Ste7 MAPK pathways^22^, while MAPK signaling also regulates filamentous growth in *C. albicans*^40^. Thus, in both yeasts, components of the pheromone signaling MAPK pathway regulate both filamentous transitions and sexual mating. Interestingly, glucose depletion has also been linked to mating in *S. pombe* and *Cryptococcus neoformans*, where a drop in glucose levels leads to increased mating efficiencies^41–43^. Together, these investigations further link glucose starvation to a conserved role in the regulation of fungal signaling pathways, including enabling sexual reproduction in multiple fungal species.

The terminal MAP kinases Cek1/2 have been shown to have overlapping roles in *C. albicans* mating, as deletion of both factors was necessary to block mating^23^. Scaduto et al. demonstrated that *CEK2* expression is lower in white cells than in opaque cells and that ectopic expression of *CEK2* (but not *CEK1*) dramatically increased the mating efficiency of white cells^17^. Here, we show that both Cek1 and Cek2 are required for glucose depletion-induced white cell mating (Fig. 2b and Table 1, crosses 1, 6, and 7), and again that ectopic expression of *CEK2* but not *CEK1* promotes this mating (Fig. 2b and Table 1, crosses 11, 12, and 13). Consistent with these observations, Cek2 protein levels (but not Cek1 levels) were increased under glucose-depleted conditions (Fig. S3), indicating that Cek2 contributes to mating in the absence of glucose. Cph1 (ortholog of *S. cerevisiae* Ste12) and Tec1 are transcription factors that act downstream of the pheromone MAPK pathway in *C. albicans* and *S. cerevisiae*^23,32–34^. In response to pheromone, *S. cerevisiae* Tec1 is phosphorylated by Fus3 (*C. albicans* Cek2 ortholog) and degraded through SCFCdc4. This pheromone-induced destruction of Tec1 ensures that MAPK signaling specifically promotes mating^32,33^. Interestingly, pheromone induces the expression of *TEC1* and enhances the formation of biofilms in white cells of *C. albicans*^34^, although the relative impact of *TEC1* differed in two independent studies^34,44^. We demonstrate that *TEC1* expression is reduced upon glucose depletion and that deletion of *TEC1* promotes white cell mating in *C. albicans* (Fig. 4a and Table 1). Conversely, ectopic expression of Tec1 repressed white cell mating (Table S2). These findings indicate that Tec1 functions as a key repressor of mating in *C. albicans* white cells.

The Dig1 transcription factor is involved in invasive/filamentous growth and mating, and functions as an inhibitor of Cph1 in *C. albicans*^36^. Although Tec1 and Efg1 are not essential for mating in *C. albicans*, they coordinate with Dig1 to regulate mating efficiency^36^. Here, we identified Dig1 as a key regulator of Cph1 and white cell mating (Figs. 2 and 4, Tables 1 and S2). Therefore, together with Cek2, Tec1, and Cph1, Dig1 functions as a key regulator of glucose depletion-induced white cell mating in *C. albicans* (Fig. 4e).

In addition to regulating mating, the white-opaque switch impacts many other aspects of *C. albicans* biology including interactions with host cells^8^. Under certain culture conditions, mating-incompetent *MTL***a**/α heterozygous cells are also capable of undergoing white-opaque switching^45^, suggesting that this epigenetic switch could have evolved for purposes other than mating. Given the remarkable differences in gene expression and metabolic profiles between white and opaque cells, this “division of labor” may benefit *C. albicans* adaptation to different host niches. Given that the white-cell type of *C. albicans* is more common than the opaque-cell type, white-cell mating could also be more frequent in nature than that of opaque cells. Thus, white cell mating may represent a more ancient mode of sexual reproduction that shows closer parallels to that of other yeasts such as *S. pombe* and *S. cerevisiae*. Our study therefore further demonstrates the diverse mating strategies of fungal species and sheds new light on the regulation of sex in *C. albicans*.

## Methods

### Strains and growth conditions

Yeast strains used in the study are listed in Table S3. *C. albicans* strains were stored at −80 °C in yeast extract-peptone-dextrose (YPD, 20 g/L peptone, 10 g/L yeast extract, 20 g/L glucose) medium plus 25% glycerol. To revive the strains from the frozen stocks, *C. albicans* cells were scratched with a sterile toothpick and streaked and grown on YPD medium plates at 30 °C. YPD and modified Lee’s glucose medium supplemented with 5 μg/mL phloxine B were used for routine growth of *C. albicans*.

YPD-K (20 g/L peptone, 10 g/L yeast extract, 20 g/L glucose, 2.5 g/L K_2_HPO_4_, 20 g/L agar, pH 7.3) and YP-K (20 g/L peptone, 10 g/L yeast extract, 2.5 g/L K_2_HPO_4_, 20 g/L agar, pH 7.3) medium plates were used for the induction of mating projections and quantitative mating assays of *C. albicans*^18^. Phloxine B and K_2_HPO_4_ were purchased from Sigma-Aldrich (Cat. Nos., P2759 and P9666; St. Louis, MO, USA). Synthetic complete dextrose (SCD) amino acid dropout media were used for selectable growth in quantitative mating assays.

### Plasmid construction

Primers used for PCRs in the study are listed in Table S4. To construct plasmids *pACTS-CEK1*, *pACTS-CEK2*, *pACTS-CPH1*, and *pACTS-DIG1*, the *CEK1*, *CEK2*, *CPH1*, and *DIG1* open reading frame (ORF) fragments were PCR amplified from *C. albicans* SC5314 genomic DNA and subcloned into the *Eco*RV/*Hind*III site of plasmid pACTS^18^. To construct plasmid *pACT1-TEC1*, the *TEC1* open reading frame (ORF) fragments were PCR amplified from *C. albicans* SC5314 genomic DNA and subcloned into the *Eco*RV/*Hind*III site of plasmid pACT1^10^. To construct the reconstituted plasmid pSFS2a-*TEC1p-TEC1*, the 3′-UTR fragment and 5′-UTR plus ORF region of *TEC1* were amplified from *C. albicans* SC5314 genome and sequentially inserted into the *Sac*II/*Sac*I and *Apa*I/*Xho*I sites of the plasmid pSFS2a^46^.

### Strain construction

To construct *C. albicans* strain GH1350α (GGB1418), the *Apa*I/*Sac*I linearized plasmid L23.14 (also named plasmid T2A-MTL, a pSFS2A-based MTL locus deletion plasmid) was transformed into strain SN250 to replace the *MTL***a** allele^45^. The resulting strain was then grown in YPmal medium (20 g/L peptone, 10 g/L yeast extract, 20 g/L maltose) for FLP-mediated excision of the *SAT1*/flipper cassette. Using a similar strategy, the *MTL***a** or *MTL*α locus was disrupted in the corresponding *MTL* heterozygous strains with ApaI/SacI linearized plasmid L23.14, generating strains SN152αu, CCJS837 (*wor1Δ/Δα*), GBS2775 (*cek1Δ/Δ***a**), GBS2776 (*cek2Δ/Δ***a**), GBS2783 (*cek1Δ/Δ*α), and GBS2784 (*cek2Δ/Δ*α).

#### Strain GBS2347

The *URA3-IRO1* cassette was amplified from *C. albicans* SC5314 genomic DNA and integrated into the original locus of strain CCJS837^15^, generating strain GBS2347.

#### Strains GBS2824 (*cek1Δ/Δ ura3Δ/Δ*a) and GBS2826 (*cek2Δ/Δ ura3Δ/Δ*a)

Stains GBS2775 and GBS2776 were streaked onto solid SCD medium containing 5-fluorooroic acid (5-FOA, A601555, Sangon Biotech, Shanghai, China) and incubated at 37 °C for 7 days for the selection of *ura3Δ/Δ* mutant. Colonies of uridine auxotrophy were confirmed by growing on solid SCD medium lacking uridine. The resulting *ura3Δ/Δ* strains were then transformed with the *ARG4* cassette amplified from SC5314 genomic DNA, generating GBS2824 and GBS2826.

#### GBS2799 (*wor1Δ/Δ*a pACTS-CEK1), GBS2809 (*wor1Δ/Δ*α pACTS-CEK1), GBS2802 (*wor1Δ/Δ*a pACTS-CEK2), GBS2810 (*wor1Δ/Δ*α pACTS-CEK2), GBS2682 (*wor1Δ/Δ*a pACTS-CPH1) and GBS2779 (*wor1Δ/Δ*α pACTS-CPH1)

To construct strains ectopically expressing *CEK1*, *CEK2*, or *CPH1*, the *Asc*I-linearized *pACTS-CEK1*, *CEK2,* or *CPH1* plasmid, respectively, was transformed into strains GH1608 and CAY3336u.

#### GBS2654 (*wor1Δ/Δ*α pACT1-TEC1) and GBS2763 (WTα pACT1-TEC1)

To construct strains ectopically expressing *TEC1*, the *Asc*I-linearized *pACT1-TEC1* plasmid was transformed into strains CCJS837 and GH1605.

#### GBS2937 (*wor1Δ/Δ*a pACTS-DIG1), GBS2931 (*wor1Δ/Δ*α pACTS-DIG1), GBS2946 (*wor1Δ/Δ*α pACTS-DIG1), and GBS2963 (WTα pACTS-DIG1)

To construct strains ectopically expressing *DIG*1, the *Asc*I-linearized *pACTS-DIG1* plasmid was transformed into strains GH1608, CAY3336u, CCJS837, and GH1350α.

#### GBS2891 (*tec1Δ/Δ*a + *TEC1p-TEC1*) and GBS2897 (*tec1Δ/Δ*α + *TEC1p-TEC1*)

To generate the *TEC1* reconstituted strain, the plasmid pSFS2a-*TEC1p-TEC1* was linearized with *Apa*I/*Sac*I and transformed into strains GH2783 and JSM115.

#### GBS2828 (*wor1Δ/Δ*a MFA1p-GFP)

To construct the *MFA1p-GFP* reporter strain, the *GFP-SAT1* cassette was amplified from the plasmid pNIM1^46^ and then transformed into strain GH1248.

#### GBS2604 (*tec1Δ/Δwor1Δ/Δ*α) and GBS2792 (*tec1Δ/Δwor1Δ/Δ*a)

To construct the *tec1Δ/Δ wor1Δ/Δ* double mutant, the first copy of *WOR1* gene in JSM115 (*tec1Δ/Δ*α)^47^ was disrupted with the *Apa*I/*Sac*I linearized plasmid pSFS2a-*WOR1* KO^45^. The resulting strain was subject to the FLP-mediated excision of the SAT1/flipper cassette by growing on YPmal medium, generating strain *tec1Δ/Δα WOR1/wor1::FRT*. To disrupt the second allele of *WOR1*, the ApaI/SacI linearized plasmid pSFS2a-*WOR1* KO was transformed into strain *tec1Δ/Δα WOR1/wor1::FRT*, generating the double mutant GBS2604. A similar strategy was used to delete the first allele of *WOR1* in strain GH2783 (*tec1Δ/Δ****a***). The resulting strain *tec1Δ/Δ****a*** *WOR1/wor1::FRT* was then transformed with the PCR product of *SAT1* cassette flanked by 60-bp 5′- and 3′-homologous sequences of *WOR1*, generating the double mutant GBS2792.

#### GBS3005 (*dig1Δ/Δ*a), GBS3009 (*dig1Δ/Δ*α), GBS2874 (*wor1Δ/Δ dig1Δ/Δ*a), and GBS2877 (*wor1Δ/Δ dig1Δ/Δ*α)

To delete both alleles of *DIG1* in the WT and *wor1Δ/Δ* mutant, fusion PCR reactions were performed^48^. PCR products for targeting the *DIG1* locus (5′- and 3′-flank regions of *DIG1*) were amplified from *C. albicans* SC5314 genomic DNA with primers GGB1289/GGB1290 and GGB1291/GGB1292, respectively. Selectable marker genes *CaURA3, CaHIS1*, *CmLEU2*, and *caSAT1* were PCR amplified from plasmids pGEM-URA3, pGEM-HIS1, pSN40, and pNIM1, respectively^46,48,49^. Fusion PCR assays were then performed using the DIG1 5′- and 3′-flank fragments and selectable marker cassettes as templates. To delete the first allele of *DIG1*, fusion PCR products containing the *CaURA3* marker were transformed into the WT strains GH1013 (WT**a**) and SN152αu (WTα). To delete the second allele of *DIG1*, fusion PCR products containing the *CaHIS1* or *CmLEU2* marker were transformed into heterozygous mutants. To generate the *dig1Δ/Δ wor1Δ/Δ* double mutant, fusion PCR products containing the *CaHIS1* or *CmLEU2* marker were transformed into strain CAY3339 (*wor1Δ/Δ***a**) or CAY3336 (*wor1Δ/Δ*α), respectively. Then, the second allele of *DIG1* was deleted using fusion PCR products containing the *caSAT1* marker.

#### LTS1161 (WTa Cph1-TAP), LTS1172 (*wor1Δ/Δ*a Cph1-TAP), LTS1338 (WTα Cph1-TAP), and LTS1209 (*wor1Δ/Δ*α Cph1-TAP)

To construct the TAP-tagged Cph1 strains, one allele of *CPH1* was first deleted using a *CaURA3* cassette flanked by 5′- and 3′-fragments *of CPH1* amplified with the fusion PCR assay as earlier described. The *CPH1-CaURA3* disruption cassette was transformed into strains GH1013, GH1608, SN152αu, and CCJS837, generating corresponding *cph1::CaURA3/CPH1* heterozygous strains. Then, the *TAP-ARG4* cassette flanked by approximately 60 bp 5′- and 3′-homologous sequences of *CPH1* was PCR amplified from strain CaLC2993^50^ with primers LTG01/LTG02 and was transformed into the *cph1::CaURA3/CPH1* heterozygous strains, generating strains in which the endogenous Cph1 protein was TAP-tagged: LTS1161, LTS1172, LTS1338, and LTS1209.

A similar strategy was used to construct the TAP-tagged Cek2, Tec1, Cek1, and Dig1 expression strains: LTS1164 (WT**a** Cek2-TAP), LTS1176 (*wor1Δ/Δ***a** Cek2-TAP), LTS1343 (WTα Cek2-TAP), and LTS1221 (*wor1Δ/Δ*α Cek2-TAP); LTS1186 (WT**a** Tec1-TAP), LTS1183 (*wor1Δ/Δ***a** Tec1-TAP), LTS1336 (WTα Tec1-TAP), and LTS1213 (*wor1Δ/Δ*α Tec1-TAP); LTS1302 (WT**a** Cek1-TAP), LTS1179 (*wor1Δ/Δ***a** Cek1-TAP), LTS1341 (WTα Cek1-TAP), LTS1217 (*wor1Δ/Δ*α Cek1-TAP), LTS1483 (*wor1Δ/Δ***a** Dig1-TAP) and LTS1480 (*wor1Δ/Δ*α Dig1-TAP).

#### LTS1463 (*wor1Δ/Δ*a Cph1-TAP *pACTS-DIG1*) and LTS1345 (*wor1Δ/Δ*a, Cph1-TAP *pACTS*)

The plasmids *pACTS-DIG1* and *pACTS* were linearized with *Asc*I and transformed into the TAP-tagged Cph1 expression strain LTS1172, generating LTS1463 and LTS1345, respectively.

#### LTS1452 (*gpr1Δ/Δarg4Δ/Δ*a) and FSR320 (*hgt12Δ/Δ ura3Δ/Δ*a)

To construct the *gpr1Δ/Δ***a** mutant, one allele of *GPR1* was deleted using a *CdHIS1* cassette flanked by 5′- and 3′-fragments *of GPR1* amplified with the fusion PCR assay as earlier described. The *GPR1-CdHIS1* disruption cassette was transformed into the WT strain SN152**a** (WT**a**), generating corresponding *gpr1::CdHIS1/GPR1* heterozygous strains. To delete the second allele of *GPR1*, fusion PCR products containing the *CmLEU2* marker were transformed into the heterozygous mutant. Similarly, to generate the *hgt12Δ/Δ***a** mutant, fusion PCR products containing the *CaARG4* marker were transformed into the WT strains GH1013 (WT**a**) to generate the heterozygous mutant and the second allele of *HGT12* was then deleted using fusion PCR products containing the *cdHIS1* marker.

### Mating assays

*C. albicans* mating assays were performed as described in previous publications with slight modifications^15^. Briefly, *C. albicans* cells were first grown on solid Lee’s glucose medium at 25 °C for 5 days. Homogeneous white or opaque cells were collected and resuspended in water at a concentration of 1 × 10^10^ cells/mL. *C. albicans* cells of the two mating partners (3 μl for each) were mixed and spotted on YP-K and YPD-K media. After 7 days of incubation at 25 °C, mating mixtures were then replated onto three types of selective SCD media lacking amino acids depending on the mating partner auxotrophies. Mating frequency = conjugants / (limiting parents + conjugants)^15^. Three biological replicates were performed for each mating cross.

### RNA extraction and quantitative real-time PCR assays

RNA extraction and quantitative real-time PCR (qRT-PCR) assays were performed according to our previous publication with slight modifications^15^. *C. albicans* strains were grown overnight in SCD liquid medium at 25 °C. Cells were washed and resuspended in ddH_2_O at a concentration of 3.3 × 10^9^ cells/mL. Three microliters of fungal cells were spotted on YP-K or YPD-K media. After 3 days of incubation at 25 °C, cells were harvested and washed with 1 × PBS. Total RNA was then extracted using RNA purification kit (Thermo scientific, K0732, Thermo scientific, Waltham, USA). For qRT-PCR assays, 1 μg of total RNA of each sample was used to synthesize cDNA with ReverAid H Minus Reverse Transcriptase (EP0451, Thermo Scientific, Waltham, USA). Quantification of transcripts was performed in Bio-Rad CFX96 real time detection system (Bio-Rad, Hercules, USA) using SYBR green master mix (QPS-201, TOYOBO, Osaka, Japan) under the condition: 95 °C for 1 min, followed by 95 °C for 15 s, 57 °C for 20 s, and 72 °C for 50, for 40 cycles. The signal of each sample was normalized to the expression level of *C. albicans ACT1*. Data were analyzed using Bio-Rad CFX Manager 3.1 for Bio-Rad Real-Time PCR system and presented as the average ± standard error (SEM). Three biological replicates were performed.

### Statistics and reproducibility

All data presented in quantitative mating and qRT-PCR assays were performed at least three independent biological replicates and analyzed using GraphPad Prism 9.0 software. For quantitative mating analyses, data were analyzed using two-sided Student’s *t*-tests. For qRT-PCR assays, data were analyzed using two-sided Student’s *t*-tests. Methods for statistical analyses are specified in the legend of every individual figure or table. Colony and cellular morphologies presented in the figures were representative images of three independent experiments.

### Proteomic assay

The *wor1*Δ/Δ**a** strains were initially grown in YPD-K medium at 25 °C for 2 days. Approximately 1 × 10^7^ cells were spotted on YP-K and YPD-K media and cultured for 3 days at 25 °C. Cells were harvested and protein extracts were prepared as described below for the western blot analyses. For LC-MS/MS analysis, proteins were digested via FASP method using Nanosep 10k filters (Pall Life Sciences, USA)^51^. After three-time buffer displacement with 8 M urea in 25 mM NH_4_HCO_3_, proteins were reduced with 10 mM DTT at 37 °C for 30 min, and followed by alkylation with 30 mM iodoacetamide at 25 °C for 45 min in dark. Then, the filter was washed with 20% acetonitrile (ACN) once followed by digestion buffer (30 mM NH_4_HCO_3_) three times. Overnight digestion was carried out using trypsin (enzyme/protein (w/w) ratio as 1:50) at 37 °C for 12 h. Then, the solution was filtrated out and the filter was washed twice with 15% ACN. All the filtrates were pooled and vacuum-dried. LC-MS/MS analysis was performed using an EASY-nLC 1200 system (Thermo Fisher Scientific, USA) coupled to an Orbitrap Fusion Lumos mass spectrometer (Thermo Fisher Scientific, USA). A one-column system was adopted for all analyses. Samples were analyzed on a home-packed C18 column (75 µm i.d. × 25 cm; ReproSil-Pur 120 C18-AQ, 1.9 µm (Dr. Maisch GmbH, Germany))^52^. The mobile phases consisted of Solvent A (0.1% formic acid) and Solvent B (0.1% formic acid in 80% ACN). The eluted peptides were sprayed directly into Orbitrap Fusion Lumos mass spectrometer. Data-dependent analysis was employed in MS analysis: The MS1 scans were acquired in Orbitrap (resolution of 60,000, 350-1600 m/z, and auto maximum ion injection time). The cycle time between master scans was set as 3 s, and the precursor ions were fragmented in HCD mode and analyzed using Orbitrap analyzer with a resolution of 15,000 and normalized collision energy (NCE) of 30%. The raw data were processed using Proteome Discoverer software (version 2.4, Thermo Fisher Scientific) with in-house Mascot search engine (version 2.7.0, Matrix Science). The data search was performed against the *C. albicans* protein database (19401, 20211129) downloaded from UniProt. Trypsin/P was chosen as the enzyme, and up to two missed cleavages were allowed. The precursor mass tolerance was set as 10 ppm and fragment mass tolerance was set as 0.05 Da. Carbamidomethylation on cysteine as a fixed modification, N-acetylation in the protein N-terminal and oxidation on methionine as variable modifications. The false discovery rate threshold was set at 0.05 and 0.01 for proteins and peptides, respectively. Three replicates were performed. Differentially enriched or expressed proteins were tested using the DEP package version 3.16.

### Western blotting

Total protein was extracted for western blot analysis according to a published protocol^18^. *C. albicans* strains were grown in YPD-K medium at 25 °C for 2 days. Approximately 1 × 10^7^ cells of each experimental strain were then spotted on YP-K and YPD-K media and cultured for 3 days at 25 °C. Cells were harvested, rinsed twice with 1 × PBS, and then resuspended in 200 μL lysis buffer (50 mM Tris-HCl, pH8.0, 150 mM NaCl, 1% NP-40, 1% Na-deoxycholate, 0.1% (w/w) SDS, 1 mM EGTA, 1 mM EDTA, 1 mM PMSF) containing the protease inhibitor mix (Cat. No. 11873580001 Roche Diagnostics, Mannheim, Germany). *C. albicans* cells were lysed with a bead beating instrument (40 s beating followed by 1 min cooling on ice for 5 cycles). The supernatant of cell lysates was collected by centrifugation at 12,000 rpm for 15 min and the protein concentration was determined by Bradford assays (Cat. No. B6916, Sigma-Aldrich, Inc). Total protein of each sample was separated by 10% SDS-PAGE gel and then transferred to a polyvinylidene difluoride (PVDF) membrane (Millipore, Bedford, MA). Membranes were washed twice for 10 min with TBS buffer (20 mM Tris-HCl, pH 7.4, 150 mM NaCl) and blocked in 5% milk in TBS buffer containing 0.05% Tween-20 (TBST) at room temperature for 2 h with gentle shaking. For anti-TAP blots, a horseradish peroxidase (HRP)-conjugated monoclonal anti-TAP antibody (1:1000 dilution, P1291, Sigma-Aldrich, Inc) was used. For anti-Cdc28 blots, a monoclonal anti-Cdc28 antibody (1:1000 dilution, sc-515762, Santa Cruz Biotechnology, Inc) was used, followed by a HRP-conjugated secondary antibody (1:2000 dilution, 7076 S, Cell Signaling Technology Inc). The immunopositive bands were visualized using an enhanced chemiluminescent substrate (32209 Thermo Scientific, USA) and analyzed using Image Lab 3.0 for a Chemidoc XRS + system (Bio-Rad).

### Reporting summary

Further information on research design is available in the Nature Portfolio Reporting Summary linked to this article.

## Supplementary information

Supplementary Information

Description of Additional Supplementary Files

Supplementary Dataset 1

Reporting Summary

## Data Availability

The mass spectrometry proteomics data have been deposited to the ProteomeXchange Consortium (http://proteomecentral.proteomexchange.org) via the iProX partner repository with the dataset identifier PXD040559. Source data are provided in this paper. Source data are provided with this paper.

## Source data

Source Data
