# Supplementary material for: Glucose depletion enables Candida albicans mating independently of the epigenetic white-opaque switch
Source: Nat Commun. 2023 Apr 12;14:2067. doi: 10.1038/s41467-023-37755-8 (PMC10097730; doi:10.1038/s41467-023-37755-8)
Supplement: Supplementary file 1 — Supplementary Information [file 41467_2023_37755_MOESM1_ESM.pdf]

Supplementary Information for

Glucose depletion enables *Candida albicans* mating independently of the epigenetic white-opaque switch

Guobo Guan[a,b,#], Li Tao[a,#], Chao Li[a,#], Ming Xu[a], Ling Liu[b], Richard J Bennett[c], and Guanghua Huang[a,d,e,*]

*Please direct all correspondence to:

Guanghua Huang

Email: huanggh@fudan.edu.cn

This PDF file includes:

Supplementary Figures S1 to S3, Tables S1 to S4

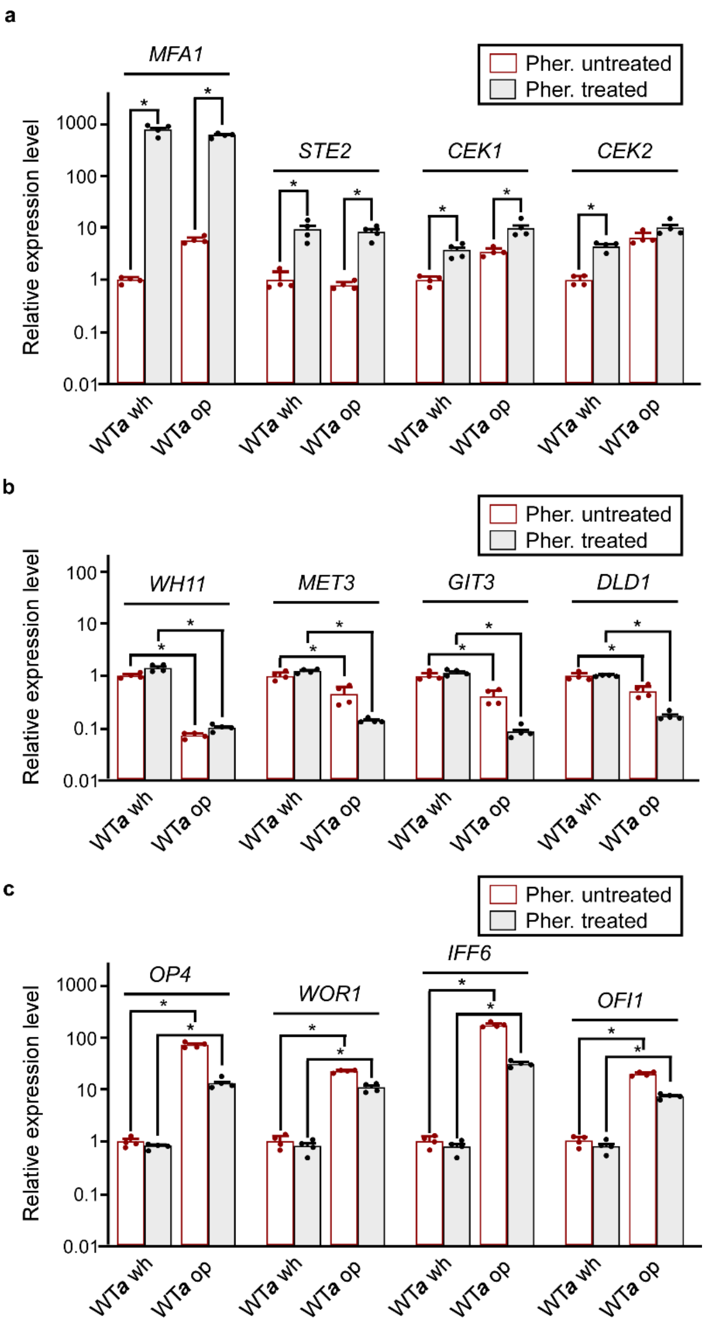

Supplementary Figure 1. Relative expression levels of mating associated genes (a), white-specific genes (b), and opaque-specific genes (c) in the WTa **white and opaque cells treated with or without α-pheromone.** Approximately 1 x 10^7 of white or opaque cells were spotted onto YP-K medium and incubated at 25°C for three days. Three μL of ddH_2O or 200 μM α-pheromone was added onto the spots every 24 hours. The relative expression level of each gene in WT**a** white cells untreated with α–pheromone (treated with ddH_2O) was set as '1'. Data are presented as the average ± SEM. This figure is associated with **Fig. 3b**.

Statistical analyses: two-sided *t* test, $n = 4$.

Panel (a): pheromone-treated vs. pheromone-untreated. WT**a** wh *MFA1*, *P* = 9.8E-05; WT**a** op *MFA1*, *P* =4.4E-07; WT**a** wh *STE2*, *P* =0.0054; WT**a** op *STE2, P* =0.0007; WT**a** wh *CEK1*, *P* =0.0040; WT**a** op *CEK1, P* =0.0103; WT**a** wh *CEK2*, *P* =0.0002; WT**a** op *CEK2, P* =0.1154).
Panel (b): white cells vs. opaque cells. *WH11:* Pher. untreated, *P* = 4.3E-06; Pher. Treated, *P* =2.6E-05. *MET3:* Pher. untreated, *P* = 0.0056; Pher. treated, *P* =3.5E-07. *GIT3*: Pher. untreated, *P* =0.0015; Pher. treated, *P* =9.6E-06. *DLD1*: Pher. untreated, *P* =0.0043; Pher. treated, *P* =1.6E-08).
Panel (c): white cells vs. opaque cells. *OP4*, Pher. untreated, *P* =2.4E-06; Pher. treated, *P* =0.0001. *WOR1*, Pher. untreated, *P* = 4.7E-07; Pher. treated, *P* =7.8E-05. *IFF6*, Pher. untreated, *P* =8.9E-07; Pher. treated, *P* =3.1E-06. *OFI1*, Pher. untreated, *P* =7.4E-07; Pher. treated, *P* =5.3E-06.

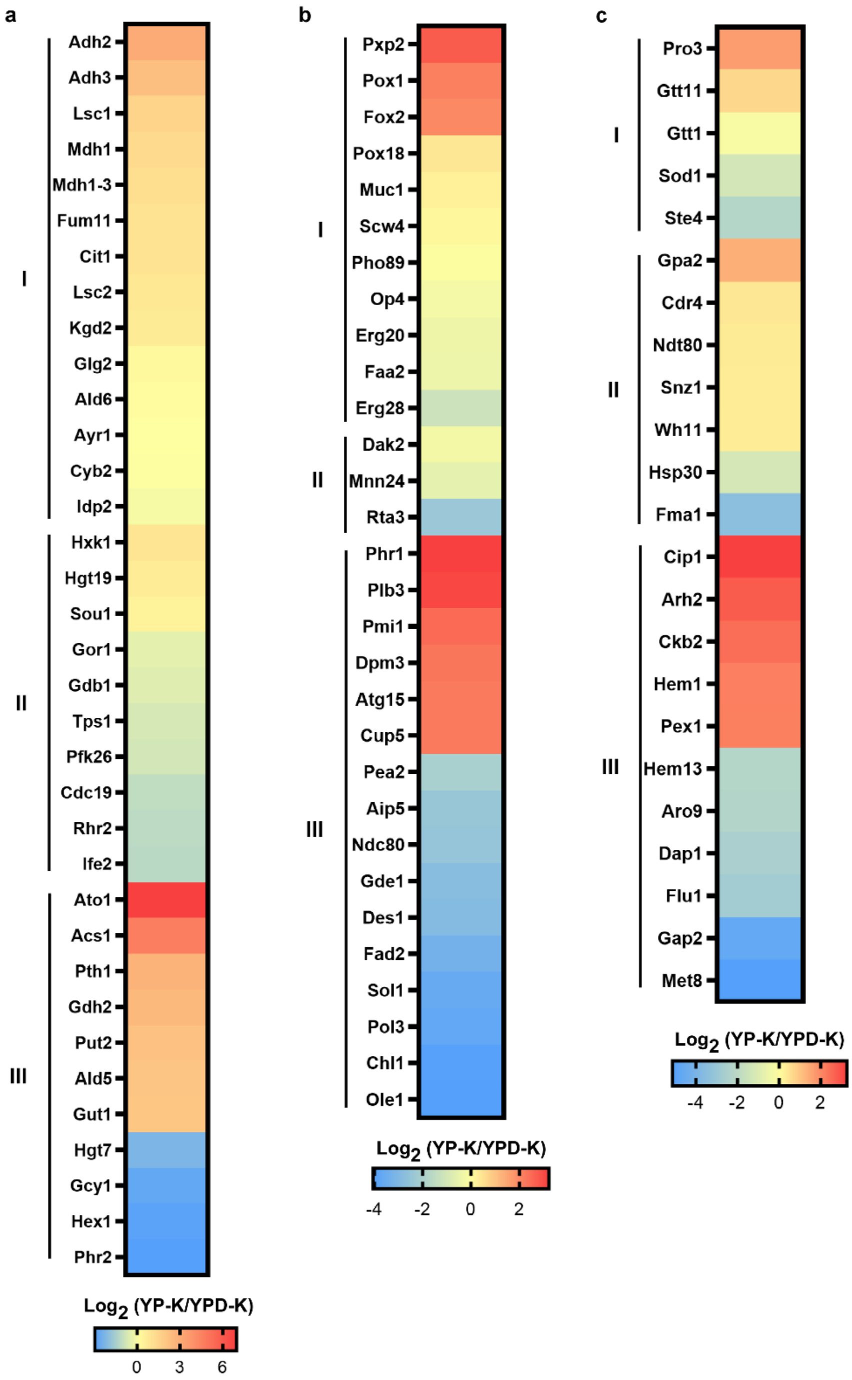

Supplementary Figure 2. Protein expression profiles of the *MTL*a *wor1*Δ/Δcells grown on YP-K and YPD-K media. The heatmaps show the expression profiles obtained from the LC-MS/MS analysis. Colors represent the relative expression levels of proteins (= Log_2 (YP-K/YPD-K), n =3. YP-K/YPD-K, mean label-free quantification (LFQ) intensity ratio (red: enriched in YP-K medium; blue: depleted in YP-K medium). Proteins categories (based on the GO enrichment analysis; **Dataset S1**): carbohydrate metabolism (a); lipid, fatty acid metabolism, and cell fate (b); and stress response, mating and signaling (c). In each panel, proteins are clustered into three groups (I, II, III) according

to the transcriptional characteristics in white and opaque cells. I, opaque-enriched; II, white-enriched; III, transcriptional expression independent of white-opaque switching (based on the data from Lan *et al.*, PNAS, 2002).

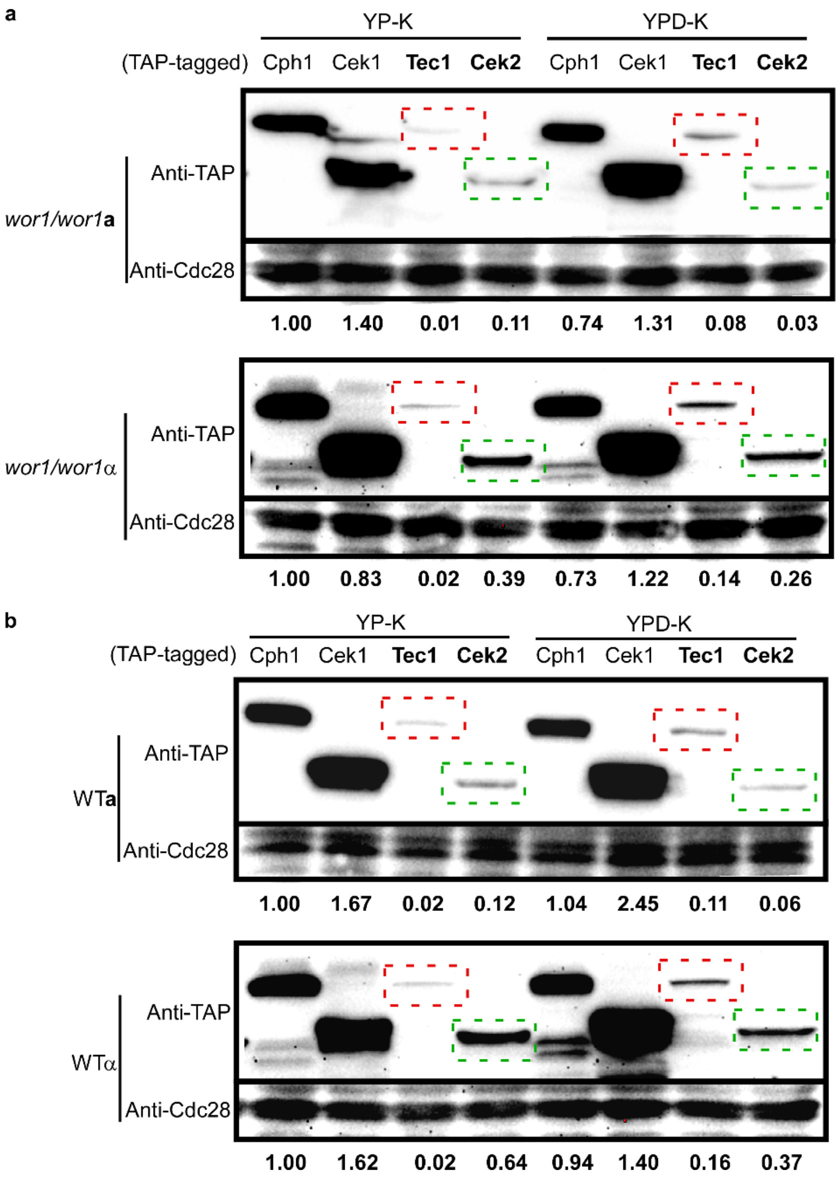

Supplementary Figure 3. Protein expression levels of the Cph1, Tec1, Cek1, and Cek2 in the *wor1/wor1* mutants (a) and WT strains (b) in YP-K and YPD-K media. The TAP-tagged genes were introduced into *wor1/wor1* and WT white strains. Cdc28 served as loading controls for Western blotting assays. The numbers indicate the relative intensities of sample bands, which were quantified by ImageJ 1.53k (Java based image-processing and analysis software, the intensity of Cdc28 served as the reference).

Supplementary Table 1. Mating frequencies of the *wor1Δ/Δ*a x *wor1Δ/Δα* cross on different culture media. Source data are provided as a Source Data file.

	Media	*wor1Δ/Δ a,*wh x*wor1Δ/Δ α,*wh
(1)	YPD-K (YP-K + 2% Glucose)	$<3.0\times10^{-9}$
(2)	YP-K	$(1.4\pm0.9)\times10^{-6*}$
(3)	Y-K	$(5.1\pm2.8)\times10^{-6*}$
(4)	P-K	$(2.6\pm1.0)\times10^{-6*}$
(5)	Agar-K	$(8.0\pm1.4)\times10^{-4*}$
(6)	YP-K+0.1% Glucose	$(1.3\pm0.3)\times10^{-6*}$
(7)	YP-K+0.5% Glucose	$(1.9\pm2.0)\times10^{-7}$
(8)	YP-K+1% Glucose	$<3.0\times10^{-8}$
(9)	YP-K+0.5% Glucose + 1 mM 2-DG	$(1.7\pm0.9)\times10^{-5*}$
(10)	YP-K+2% Mannitol	$<1.4\times10^{-8}$
(11)	YP-K+2% Galactose	$(5.4\pm2.6)\times10^{-7}$
(12)	YP-K+2% Glycerol	$(6.1\pm5.7)\times10^{-7}$
(13)	YP-K+2% Sodium acetate	$<2.8\times10^{-8}$
(14)	YP-K+2% Oleinic acid	$(9.8\pm4.0)\times10^{-7*}$
(15)	YP-K+0.074M saccinic acid	$<4.4\times10^{-8}$

Notes:

The mixture of "**a**" and α mating partners (~3 x 10^7 cells for each strain) were spotted on different media and cultured at 25°C for seven days. The mating mixture was then replated onto SCD selection media to determine mating frequencies. n = 3 independent experiments, and the results represent the average ± SD. "<" indicates no progeny colonies observed. Statistical differences were determined by two-sided unpaired Student's *t*-test, * = $P < 0.05$. Compared to the mating efficiency on YPD-K medium (1), *P* value are: YP-K (2), $P = 0.0437$; Y-K (3), $P = 0.0077$; P-K (4), $P = 0.0098$; agar-K (5), $P = 0.0005$; 0.1% Glucose (6), $P = 0.0010$; 0.5% Glucose (7), $P = 0.1886$; 0.5% Glucose + 1 mM 2-DG (9), $P = 0.0353$; 2% Galactose (11), $P = 0.1464$; 2% Glycerol (12), $P = 0.1372$; 2% Oleinic acid (14), $P = 0.0136$. Strains used： *wor1Δ/Δ***a**, GH1248; *wor1Δ/Δ*α, GBS2347.

Media:

(1) YPD-K: 20 g/L peptone, 10 g/L yeast extract, 20 g/L glucose, 2.5 g/L K_2HPO_4, 20 g/L agar;
(2) YP-K: 20 g/L peptone, 10 g/L yeast extract, 2.5 g/L K_2HPO_4, 20 g/L agar;
(3) Y-K: 10 g/L yeast extract, 2.5 g/L K_2HPO_4, 20 g/L agar;
(4) P-K: 20 g/L peptone, 2.5 g/L K_2HPO_4, 20 g/L agar;
(5) Agar-K: 2.5 g/L K_2HPO_4, 20 g/L agar;

(6) YP-K+0.1% Glucose: 20 g/L peptone, 10 g/L yeast extract, 1 g/L glucose, 2.5 g/L K_2HPO_4, 20 g/L agar;
(7) YP-K+0.5% Glucose: 20 g/L peptone, 10 g/L yeast extract, 5 g/L glucose, 2.5 g/L K_2HPO_4, 20 g/L agar;
(8) YP-K+1% Glucose: 20 g/L peptone, 10 g/L yeast extract, 10 g/L glucose, 2.5 g/L K_2HPO_4, 20 g/L agar;
(9) YP-K+0.5% Glucose + 1 mM 2-DG: 20 g/L peptone, 10 g/L yeast extract, 5 g/L glucose, 1 mM 2-DG, 2.5 g/L K_2HPO_4, 20 g/L agar;

(10) YP-K+2% Mannitol: 20 g/L peptone, 10 g/L yeast extract, 20 g/L mannitol, 2.5 g/L K_2HPO_4, 20 g/L agar;
(11) YP-K+2% Galactose: 20 g/L peptone, 10 g/L yeast extract, 20 g/L galactose, 2.5 g/L K_2HPO_4, 20 g/L agar;
(12) YP-K+2% Glycerol: 20 g/L peptone, 10 g/L yeast extract, 20 g/L glycerol, 2.5 g/L K_2HPO_4, 20 g/L agar;
(13) YP-K+2% Sodium acetate: 20 g/L peptone, 10 g/L yeast extract, 20 g/L sodium acetate, 2.5 g/L K_2HPO_4, 20 g/L agar;
(14) YP-K+2% Oleinic acid: 20 g/L peptone, 10 g/L yeast extract, 20 g/L oleinic acid, 2.5 g/L K_2HPO_4, 20 g/L agar;
(15) YP-K+0.074 M saccinic acid: 20 g/L peptone, 10 g/L yeast extract, 8.75 g/L succinic acid, 2.5 g/L K_2HPO_4, 20 g/L agar.

Supplementary Table 2. Mating frequencies of the WT and mutated strains of *C. albicans* on YP-K and YPD-K media. Source data are provided as a Source Data file.

Cross		YP-K	YPD-K	Fold change (YP-K/YPD-K)
(16)	*WT***a**,op **x** *WT*α,wh	$(3.9±1.7)×10^{-4}$	$(2.4±0.7)×10^{-6}$	163
(17)	*wor1Δ/Δ* **a**,wh **x** *WT*α,op	$(1.2±0.7)×10^{-3}$	$(3.7±0.6)×10^{-6}$	324
(18)	*tec1Δ/Δ TEC1p-TEC1***a**, wh **x** *tec1Δ/Δ TEC1p-TEC1*α, wh	$(1.4±0.3)×10^{-6}$	$(2.9±2.4)×10^{-8}$	48
(19)	*WT***a**,op **x** *wor1Δ/Δ pACTS*α,wh	$(5.9±2.6)×10^{-5}$	$(4.0±4.8)×10^{-8}$	1,475
(20)	*WT***a**,op **x** *wor1Δ/Δ pACT1-TEC1*α,wh	$<1.1×10^{-8*}$	$<7.5×10^{-9}$	NA
(21)	*wor1Δ/Δ* **a**,wh **x** *wor1Δ/Δ pACTS*α,wh	$(1.5±0.9)×10^{-7}$	$<3.9×10^{-9}$	>38
(22)	*wor1Δ/Δ* **a**,wh **x** *wor1Δ/Δ pACT1-TEC1*α,wh	$<4.1×10^{-9*}$	$<1.0×10^{-9}$	NA
(23)	*WT***a**,op *x WT pACTS*α,op	$(1.6±0.7)×10^{-1}$	$(6.1±0.5)×10^{-2}$	3
(24)	*WT***a**,op **x** *WT pACT1-TEC1*α,op	$(3.2±2.2)×10^{-3*}$	$(5.5±2.4)×10^{-3*}$	0.6
(25)	*WT***a**,op **x** *wor1Δ/Δ pACTS-DIG1*α,wh	$(6.8±3.1)×10^{-7*}$	$3.5×10^{-8}$	19
(26)	*wor1Δ/Δ* **a**,wh **x** *wor1Δ/Δ pACTS-DIG1*α,wh	$<7.7×10^{-9}$	$<1.3×10^{-8}$	NA
(27)	*WT***a**,op **x** *WT pACTS -DIG1*α,op	$(3.9±0.0)×10^{-2*}$	$(7.2±0.0)×10^{-3*}$	5
(28)	*dig1Δ/Δ* **a**,op **x** *dig1Δ/Δ* α,op	$(8.4±1.8)×10^{-1*}$	$(3.2±1.7)×10^{-1}$	2.6
(29)	*WT***a**,wh **x** *cek1Δ/Δ*α,wh	$<1.8×10^{-9*}$	$<1.1×10^{-9}$	NA
(30)	*WT***a**,wh **x** *cek2Δ/Δ*α,wh	$<2.2×10^{-9*}$	$<1.2×10^{-9}$	NA
(31)	*ste2Δ/Δ***a**,wh **x** *WT*α,wh	$<3.2×10^{-9*}$	$<3.0×10^{-9}$	NA
(32)	*gpr1Δ/Δ***a**, wh **x** *wor1Δ/Δ*α,wh	$(1.4±0.1)×10^{-5*}$	$(2.3±1.8)×10^{-8}$	609
(33)	*hgt12Δ/Δ***a**,wh **x** *wor1Δ/Δ*α,wh	$(3.6±3.5)×10^{-6}$	$(7.5±0.9)×10^{-8*}$	48

Notes: This table is associated with **Table 1** in the main text. Some additional controls, crosses of reconstituted strains, and opaque cells are presented here. The mixture of "**a**" and α mating partners (~3 x 10^7 cells for each strain) were spotted and cultured on YP-K and YPD-K media at 25°C for seven days. The mating mixture was then replated onto SCD selection media to determine mating frequency. *n* = 2-5 independent experiments. The results represent the average ± SD. "<" indicates no progeny colonies observed; wh, white cells; op, opaque cells. Statistical differences were determined by two-sided unpaired Student's *t*-test, * = $P < 0.05$.

Under YP-K medium: cross (19) vs. cross (20), $P = 0.0174$; cross (21) vs. cross (22), $P = 0.0467$; cross (23) vs. cross (24), $P = 0.0206$; cross (19) vs. cross (25), $P = 0.0181$; cross (21) vs. cross (26), $P = 0.0501$; cross (23) vs. cross (27), $P = 0.0469$; cross (28) vs. cross (3), $P = 0.0118$; cross (29) vs. cross (1), $P = 0.0162$; cross (30) vs. cross (1), $P = 0.0162$; cross (31) vs. cross (1), $P = 0.0162$; cross (32) vs. cross (1), $P < 0.0001$; cross (33) vs. cross (1), $P = 0.6184$.

Under YPD-K medium: cross (19) vs. cross (20), $P = 0.3132$; cross (23) vs. cross (24), $P < 0.0001$; cross (19) vs. cross (25), $P = 0.9195$; cross (23) vs. cross (27), $P < 0.0001$; cross (28) vs. cross (3), $P = 0.0269$; cross (32) vs. cross (1), $P = 0.1841$; cross (33) vs. cross (1), $P = 0.0002$.

*Autotrophic strains used for 33 crosses as described in **Table 1** and **Table S2**:

(1) WT**a**, GH1013 (*ura3Δ/Δ*); WTα, GH1350α (*arg4Δ/Δ*);
(2) WT**a**, GH1013 (*ura3Δ/Δ*); WTα, GH1350α (*arg4Δ/Δ*);
(3) WT**a**, GH1013 (*ura3Δ/Δ*); WTα, GH1350α (*arg4Δ/Δ*);
(4) WT**a**, GH1013 (*ura3Δ/Δ*); *wor1Δ/Δ*α, GBS2347, the two alleles of *WOR1* were deleted in SN152, *arg4Δ/Δ*;
(5) *wor1Δ/Δ***a**, GH1248, the two alleles of *WOR1* were deleted in GH1013, *ura3Δ/Δ*;*wor1Δ/Δ*α, GBS2347, the two alleles of *WOR1*were deleted in SN152, *arg4Δ/Δ*;
(6) *cek1Δ/Δ***a**, GBS2824 (*ura3Δ/Δ*); *cek1Δ/Δ*α, GBS2783 (*arg4Δ/Δ*);
(7) *cek2Δ/Δ***a**, GBS2826 (*ura3Δ/Δ*); *cek2Δ/Δ*α, GBS2784 (*arg4Δ/Δ*);
(8) *WT***a***, wh* (*leu2Δ/Δ*); *cek1Δ/Δcek2Δ/Δ*α, GH1247 (*ura3Δ/Δ*);
(9) *tec1Δ/Δwor1Δ/Δ***a**, GBS2792 (*leu2Δ/Δ*); *tec1Δ/Δwor1Δ/Δ*α, GBS2604 (*arg4Δ/Δ*);
(10) *wor1Δ/Δdig1Δ/Δ***a**, GBS2874 (*leu2Δ/Δ*);*wor1Δ/Δ dig1Δ/Δ*α, GBS2877 (*his1Δ/Δ*);
(11) *wor1Δ/Δ***a** *pACTS*, GBS2795 (*arg4Δ/Δ*); *wor1Δ/Δ*α*pACTS*, GBS2804 (*his1Δ/Δleu2Δ/Δ*);
(12) *wor1Δ/Δ***a** *pACTS-CEK1*, GBS2799 (*arg4Δ/Δ*); *wor1Δ/Δ*α *pACTS-CEK1*, GBS2809 (*his1Δ/Δleu2Δ/Δ*);
(13) *wor1Δ/Δ***a** *pACTS-CEK2,*GBS2802 (*arg4Δ/Δ*); *wor1Δ/Δ*α *pACTS-CEK2*, GBS2810 (*his1Δ/Δleu2Δ/Δ*);
(14) *wor1Δ/Δ***a***pACTS-CPH1*, GBS2682 (*arg4Δ/Δ*); *wor1Δ/Δ*α*pACTS-*

CPH1, GBS2779 (*his1Δ/Δleu2Δ/Δ*);

(15) *wor1Δ/Δ***a** *pACTS-DIG1*, GBS2937 (*arg4Δ/Δ*);*wor1Δ/Δ*α *pACTS-DIG1*, GBS2931 (*his1Δ/Δleu2Δ/Δ*)；

(16) WT**a**, GH1013 (*ura3Δ/Δ*); WTα, GH1350α (*arg4Δ/Δ*);

(17) *wor1Δ/Δ***a**, GH1248, the two alleles of*WOR1*were deleted in GH1013, *ura3Δ/Δ*; WTα, GH1350α (*arg4Δ/Δ*);

(18) *tec1Δ/Δ***a** + *TEC1p-TEC1*: GBS2891 (*leu2Δ/Δhis1Δ/Δ*);*tec1Δ/Δ*α+ *TEC1p-TEC1*: GBS2897 (*arg4Δ/Δleu2Δ/Δ*);

(19) WT**a**, GH1013 (*ura3Δ/Δ*); *wor1Δ/Δ*α *pACTS*, GBS2649 (arg4*Δ/Δ*);

(20) WT**a**, GH1013 (*ura3Δ/Δ*); *wor1Δ/Δα pACT1-TEC1*, GBS2654 (*arg4Δ/Δ*);

(21) *wor1Δ/Δ***a**, GH1248, the two alleles of*WOR1*were deleted in GH1013, *ura3Δ/Δ*; *wor1Δ/Δ*α*pACTS*, GBS2649 (arg4*Δ/Δ*);

(22) *wor1Δ/Δ***a**, GH1248, the two alleles of*WOR1*were deleted in GH1013, *ura3Δ/Δ*; *wor1Δ/Δα pACT1-TEC1*, GBS2654 (*arg4Δ/Δ*);

(23) WT**a**, GH1013 (*ura3Δ/Δ*); *WTα pACTS*, GBS2758 (*his1Δ/Δ*);

(24) WT**a**, GH1013 (*ura3Δ/Δ*); *WTα pACT1-TEC1*,GBS2763 (*his1Δ/Δ*);

(25) WT**a**, GH1013 (*ura3Δ/Δ*);*wor1Δ/Δα pACTS-DIG1*, GBS2946 (*arg4Δ/Δ*);

(26) *wor1Δ/Δ***a**, GH1248, the two alleles of *WOR1* were deleted in GH1013, *ura3Δ/Δ*; *wor1Δ/Δα pACTS-DIG1*, GBS2946 (*arg4Δ/Δ*);

(27) WT**a**, GH1013 (*ura3Δ/Δ*);*WTα pACTS-DIG1*, GBS2963 (*arg4Δ/Δ*).

(28) *dig1Δ/Δ***a**,GBS3005 (*arg4Δ/Δ*); *dig1Δ/Δα,* GBS3009 (*his1Δ/Δ*).

(29) *WT***a***, wh* (*leu2Δ/Δ*); *cek1Δ/Δ*α, GBS2783 (*arg4Δ/Δ*);

(30) *WT***a***, wh* (*leu2Δ/Δ*); *cek2Δ/Δ*α, GBS2784 (*arg4Δ/Δ*);

(31) *ste2Δ/Δ***a**, LWH39 (*leu2Δ/Δ*); WTα, GH1350α (*arg4Δ/Δ*);

(32) *gpr1Δ/Δ***a**, LTS1452 (*arg4Δ/Δ*); *wor1Δ/Δ*α, CAY3336 (*his1Δ/Δ, leu2Δ/Δ*);

(33) *hgt12Δ/Δ***a**, FSR320 (*ura3Δ/Δ*); *wor1Δ/Δ*α, CAY3336 (*his1Δ/Δ, leu2Δ/Δ*).

Supplementary Table 3. Strains used in this study

Strain name	Parent strain	Genotype	Usage	Reference
GH1013	CAI4	*MTL**a**/**a** ura3::imm434/ura3::imm434 his1::hisG/his1::hisG arg4::hisG/arg4::hisG*		[1]
GH1013u	GH1013	*MTL**a**/**a** ura3::imm434/ura3::imm434 his1::hisG/his1::hisG::HIS1 arg4::hisG/arg4::hisG::ARG4*	Figure 1,2,3, S1 Table 1, S2,3	This study
SN250	CAI4	*MTL**a**/α ura3::imm434::URA3-IRO1/ura3::imm434 arg4::hisG/arg4::hisG his1::hisG/his1::hisG leu2::hisG::CdHIS1/leu2::hisG::CmLEU2*		[2]
GH1350α	SN250	*MTLα/mtl**a**::FRT ura3::imm434::URA3-IRO1/ura3::imm434 arg4::hisG/arg4::hisG his1::hisG/his1::hisG leu2::hisG::CdHIS1/leu2::hisG::CmLEU2*	Figure2, Table 1, S2	This study
GH1248	GH1013	As GH1013, but *wor1::ARG4/wor1::HIS1*	Figure 1,2, 3, 4, S1,S2 Table 1, S1,2	[3]
SN152	CAI4	As CAI4, but *URA3/ura3::imm434 iro1::IRO1/iro1::imm434 his1 ::hisG/his1::hisG leu2/leu2 arg4/arg4*		[4]
SN152a	SN152	As SN152, but *his1::hisG/his1::hisG::HIS1 arg4/arg4::ARG4 MTL**a**/mtlα::FRT*	Figure 2 Table 1	This study
GH1605	SN152	As SN152, but *ura3::imm434/ura3::imm434*		[5]
SN152αu	GH1605	As SN152, but *ura3::imm434/ura3::imm434 mtl**a**::FRT/MTLα*		This study
GH1607	SN152	As GH1605, but *wor1::HIS1/wor1::LEU2*		[5]
CCJS837	SN152	As SN152, but *MTLα/mtl**a**::FRT-SAT1-FRT ura3::imm434/ura3::imm434 wor1::HIS1/wor1::LEU2*		[5]
GBS2347	CCJS837	As CCJS837, but *ura3::imm434::URA3-IRO1/ura3::imm434*	Figures 2, 4; Tables 1, S1	This study
GH1608	SN152	As SN152, but *ura3::imm434/ura3::imm434 wor1::HIS1/wor1::LEU2 MTL**a**/mtlα::FRT-SAT1-FRT*		[5]
GBS2795	GH1608	As GH1608, but *pACTS*	Figure 2 Table 1	This study

CAY3336		*α/α, ura3::imm434::URA3/ura3::imm434 iro1::IRO1/iro1::imm434 his1::hisG/his1::hisG leu2/leu2, wor1::FRT/wor1::FRT*		[6]
CAY3336u	CAY3336	As CAY3336, but *ura3::imm434/ura3::imm434*		This study
GBS2804	CAY3336u	As CAY3336u, but *pACTS*	Figure 2 Table 1	This study
GBS2799	GH1608	As GH1608, but *pACTS-CEK1*	Figure 2 Table 1	This study
GBS2809	CAY3336u	As CAY3336u, but *pACTS-CEK1*	Figure 2 Table 1	This study
GBS2802	GH1608	As GH1608, but *pACTS-CEK2*	Figure 2 Table 1	This study
GBS2810	CAY3336u	As CAY3336u, but *pACTS-CEK2*	Figure 2 Table 1	This study
cek1/cek1	SN152	As SN152, but *cek1::HIS1/cek1::LEU2*		[2]
GBS2775	SN152	As SN152, but *cek1::HIS1/cek1::LEU2 MTL**a**/mtl α::FRT-SAT1-FRT*		This study
GBS2783	SN152	As SN152, but *cek1::HIS1/cek1::LEU2 MTLα/mtl **a**::FRT-SAT1-FRT*	Figure 2 Table 1	This study
GBS2824	SN152	As SN152, but *cek1::HIS1/cek1::LEU2 arg4/arg4::ARG4 ura3::imm434/ura3::imm434 MTL**a**/mtlα::FRT-SAT1-FRT*	Figure 2 Table 1	This study
cek2/cek2	SN152	As SN152, but *cek2::HIS1/cek2::LEU2*		[2]
GBS2776	SN152	As SN152, but *cek2::HIS1/cek2::LEU2 MTL**a** /mtlα::FRT-SAT1-FRT*		This study
GBS2784	SN152	As SN152, but *cek2::HIS1/cek2::LEU2 MTLα/mtl **a**::FRT-SAT1-FRT*	Figure 2 Table 1	This study
GBS2826	SN152	As SN152, but *cek2::HIS1/cek2::LEU2 arg4/arg4::ARG4 ura3::imm434/ura3::imm434 MTL**a**/mtlα::FRT-SAT1-FRT*	Figure 2 Table 1	This study
GH1247	CAI4	As CAI4, but *MTLα/α, ura3::imm434/ura3::imm434 cek1::hisG/cek1::hisG cek2::hisG/cek2::hisG*	Figure 2 Table 1	[7]
GBS2682	GH1608	As GH1608, but *pACTS-CPH1*	Figures 2,4 Table 1	This study
GBS2779	CAY3336	As CAY3336, but *pACTS-CPH1*	Figure 2 Table 1	This study
GH2783	SN152	As SN152, but *MTL**a**/mtlα::FRT tec1::HIS1/tec1::ARG4*		[8]
JSM115	SN152	As SN152, but *tec1::HIS1/tec1::LEU2 MTLα/MTLα*		[9]
GBS2792	GH2783	As GH2783, but *wor1::FRT/wor1::FRT-SAT1-FRT*	Figure 2	This study

			Table 1	
GBS2604	JSM115	As JSM115, but *wor1::FRT/wor1::SAT1*	Figure 2 Table 1	This study
GBS2891	GH2783	As GH2783, but *tec1::HIS1::TEC1/tec1::ARG4*	Table S2	This study
GBS2897	JSM115	As JSM115, but *tec1::HIS1/tec1::LEU2::TEC1*	Table S2	This study
CAY3339		***a/a****, ura3::imm434::URA3/ura3::imm434 iro1::IRO1/iro1::imm434 his1::hisG/his1::hisG leu2/leu2, wor1::FRT/wor1::FRT*	Figure 4	[6]
GBS2874	CAY3339	As CAY3339, but *dig1::HIS1/dig1::SAT1*	Figure 2,4 Table 1	This study
GBS2877	CAY3336	As CAY3336, but *dig1::LEU2/dig1::SAT1*	Figure 2 Table 1	This study
GBS3005	GH1013	As GH1013, but *dig1::URA3/dig1::HIS1*	Table S2	This study
GBS3009	SN152αu	As SN152αu, but *dig1::URA3/dig1::LEU2*	Table S2	This study
GBS2937	GH1608	As GH1608, but *pACTS-DIG1*	Figure 2 Table 1	This study
GBS2931	CAY3336u	As CAY3336u, but *pACTS-DIG1*	Figure 2 Table 1	This study
MFA1p GFP	GH1013	As GH1013, but *MFA1/MFA1::MFA1p-GFP*	Figure 3	[10]
GBS2828	GH1248	As GH1248, but *MFA1/MFA1::MFA1p-GFP*	Figure 3	This study
LTS1161	GH1013	As GH1013, but *cph1:: URA3/CPH1::CPH1p-CPH1-TAP-ARG*	Figure S3	This study
LTS1164	GH1013	As GH1013, but *cek2:: URA3/CEK2::CEK2p-CEK2-TAP-ARG*	Figure S3	This study
LTS1186	GH1013	As GH1013, but *tec1:: URA32/TEC1::TEC1p-TEC1-TAP-ARG*	Figure S3	This study
LTS1302	GH1013	As GH1013, but *cek1:: URA3/CEK1::CEK1p-CEK1-TAP-ARG*	Figure S3	This study
LTS1172	GH1608	As GH1608, but *cph1:: URA3/CPH1::CPH1p-CPH1-TAP-ARG*	Figure S3	This study
LTS1176	GH1608	As GH1608, but *cek2:: URA3/CEK2::CEK2p-CEK2-TAP-ARG*	Figure S3	This study
LTS1179	GH1608	As GH1608, but *cek1:: URA3/CEK1::CEK1p-CEK1-TAP-ARG*	Figure S3	This study
LTS1183	GH1608	As GH1608, but *tec1:: URA3/TEC1::TEC1p-TEC1-TAP-ARG*	Figure S3	This study
LTS1483	GH1608	As GH1608, but *dig1:: URA3/DIG1::DIG1p-DIG1-TAP-ARG*	Figure 4	This study
LTS1345	LTS1172	As LTS1172, but *pACTS*	Figure 4	This study
LTS1463	LTS1172	As LTS1172, but *pACTS-DIG1*	Figure 4	This study
LTS1338	SN152αu	As SN152αu, but *cph1:: URA3/CPH1::CPH1p-CPH1-TAP-ARG*	Figure S3	This study
LTS1343	SN152αu	As SN152αu, but *cek2:: URA3/CEK2::CEK2p-*	Figure S3	This study

		CEK2-TAP-ARG		
LTS1341	SN152αu	As SN152αu, but *cek1:: URA3/CEK1::CEK1p-CEK1-TAP-ARG*	Figure S3	This study
LTS1336	SN152αu	As SN152αu, but *tec1:: URA3/TEC1::TEC1p-TEC1-TAP-ARG*	Figure S3	This study
LTS1209	CCJS837	As CCJS837, but *cph1:: URA3/CPH1::CPH1p-CPH1-TAP-ARG*	Figure S3	This study
LTS1221	CCJS837	As CCJS837, but *cek2:: URA3/CEK2::CEK2p-CEK2-TAP-ARG*	Figure S3	This study
LTS1213	CCJS837	As CCJS837, but *tec1:: URA3/TEC1::TEC1p-TEC1-TAP-ARG*	Figure S3	This study
LTS1217	CCJS837	As CCJS837, but *cek1:: URA3/CEK1::CEK1p-CEK1-TAP-ARG*	Figure S3	This study
LTS1480	CCJS837	As CCJS837, but *dig1:: URA3/DIG1::DIG1p-DIG1-TAP-ARG*	Figure 4	This study
GBS2649	CCJS837	As CCJS837, but *pACTS*	Table S2	This study
GBS2654	CCJS837	As CCJS837, but *pACT1-TEC1*	Table S2	This study
GBS2946	CCJS837	As CCJS837, but *pACTS-DIG1*	Table S2	This study
GBS2758	GH1605	As GH1605, but *ura3::imm434/ura3::imm434 his1::hisG/his1::hisG leu2/leu2::LEU2 arg4/arg4::ARG4 MTLα/mtl***a***::FRT pACTS*	Table S2	This study
GBS2763	GH1605	As GH1605, but *ura3::imm434/ura3::imm434 his1::hisG/his1::hisG leu2/leu2::LEU2 arg4/arg4::ARG4 MTLα/mtla::FRT pACT1-TEC1*	Table S2	This study
GBS2963	GH1350α	As 1350α, but *pACTS-DIG1*	Table S2	This study
LWH39	SN152	As SN152, but *MTL***a***/mtlα::FRT ste2::HIS1/ste2::ARG4*	Table S2	[8]
LTS1452	SN152**a**	As SN152**a**, but *gpr1::LEU2/gpr1::HIS1*	Table S2	This study
FRS320	GH1013	As GH1013, but *hgt12:: ARG4/hgt12:: HIS1*	Table S2	This study

Table S4. Primers used in this study

Primer number	Description	Sequence (5' to 3')	Purpose
GGB114	Wor1-5DR	GTACACTGACATCTCAAACATCAAAGATACACTTATTTCAAGTTCAATAGTGAAGTTTCAgttttcccagtcacgacgtt	For *WOR1* KO
GGB115	Wor1-3DR	AACAATCTTACCAACAGTAAGTATAATCATCTAAACCTATAGTACAACACAACATACACCtgtggaattgtgagcggata	
GGB116	5-detect fwd (wor1)	TATTGCAGCAACAGATTTCCAC	*WOR1* deletion confirmation
GGB119	3-detect rev (wor1)	CAAGGCGTCATCATATCATTC	
GGB42	5-detect (pSFS2a)	CAATGAAATCCAGACAGTCGAG	
GGB43	3-detect (pSFS2a)	CGATTAGAGACACAAACGAAC	
GGB120	Wor1 (check) fwd	CAACAACAACAACAACAACACC	
GGB121	Wor1 (check) rev	TGCCATTACCACCACTAACAC	
BJG01	URA3-IRO1 fwd	TGGAACTGCACACTACTACT	Complemented the *URA3-IRO* gene
BJG02	URA3-IRO1 rev	TCACTTCTCCTACTCCGCAAC	
GGB943	URA3-IRO1 5-detect	TTGCAAATTCTGCTACTGGAG	Confirmation of *URA3-IRO* gene
LT36	Ura3-iro1 check left	atgggtggacaagaagaagg	
GGB944	URA3-IRO1 3-detect	ACAATAGTTTCTGAGAGTGGG	
BJG03	Ura3-iro1 check right	GTTGTTGAAATTGGAATCTTG	
GGB285	Arg4-com fwd	GGACAGAAAGTTATTGCACAG	Complemented the *ARG4* gene
GGB286	Arg4-com rev	CTAGTATTGTAGTACAAGGTATC	
GGB283	His1-com fwd	AGCGGTTGTGAGAATTGACTC	Complemented the *HIS1* gene
GGB284	His1-com rev	GAGTTATCTCCAAAGGCAACAG	
GGB1210	CEK1 orf fwd-EcoRV	CCAGATATCAATATAGATCA	For construction

		ACATCATCAGCTTC	of *pACTS-CEK1* expression plasmid
GGB1211	CEK1 orf rev-HindIIII	TGAAAGCTTAACGACGACA ACCATCACC	
GGB1212	CEK2 orf fwd-EcoRV	CCAGATATCAAGAAATCTAC TGGCCCCACTC	For construction of *pACTS-CEK2* expression plasmid
GGB1213	CEK2 orf rev-HindIIII	TGAAAGCTTCTGCTGTGTTC TTAGGTCTAG	
CCJ631	CPH1 orf fwd-StuI	ATTATAAGGCCTTCAATTAC TAAAACATACAATGG	For construction of *pACTS-CPH1* expression plasmid
CCJ632	CPH1 orf rev-HindIIII	ATTAATAAGCTTACTGTATA TCATGAATGTGTTC	
GGB185	Tec1 orf fwd-StuI	AatcttaggcctATGATGTCGCAA GCTACTC	For construction of *pACT-TEC1* expression plasmid
GGB186	Tec1 orf rev-HindIIII	AatcttaagcttTGAACAAGATGA ACCAAAGC	
GGB1188	Tec1-com up fwd	TATGGGCCCATACCTCACA CCAACTCAC	For construction of *TEC1* complemented plasmid
GGB1189	Tec1-com up rev	CACGGCGCGCCTAGCAGC GGCTCGAGTGAACAAGATG AACCAAAGC	
GGB1190	Tec1-com down fwd	GTCAGCGGCCGCATCCCTG CCCGCGGGTGGAGGTTAT TGAGCAT	
GGB1191	Tec1-com down rev	TACGAGCTCGCTTGCCTGT TTGACATAG	
GGB1289	Dig1 upstream fwd	TATGGGCCCCCCACTTCAC AACAACACCAAC	Fusion PCR for *DIG1* KO
GGB1290	Dig1 upstream rev	CACGGCGCGCCTAGCAGC GGCTCGAGGGAACTATGGT CGTGTTGGTG	
LT31	pGEM maker fwd	CCGCTGCTAGGCGCGCCGT Ggttttcccagtcacgacgtt	
LT32	pGEM maker rev	GCAGGGATGCGGCCGCTG ACtgtggaattgtgagcggata	
LT2	Cm LEU2 maker fwd	CCGCTGCTAGGCGCGCCGT Gaccagtgtgatggatatctgc	
LT5	Cm LEU2 maker fwd	GCAGGGATGCGGCCGCTG ACagctcggatccactagtaacg	
LT391	SAT1 maker fwd	CCGCTGCTAGGCGCGCCGT GCCATCATAAAATGTCGAG CGTC	
LT392	SAT1 maker rev	GCAGGGATGCGGCCGCTG ACTGCAGGACCACCTTTGA TTG	

GGB1291	Dig1 downstream fwd	GTCAGCGGCCGCATCCCTGCCCGCGGCCAGCATATTATTGTTGCTC	
GGB1292	Dig1 downstream rev	TACGAGCTCGAACAAGGGTAGATAAGAAGC	
GGB83	5-detect (his1)	TTGTCTTGCTGTCCTATTGC	Confirmation of selectable maker
GGB142	3-detect (his1)	TAGCTTGGCGTAATCATGGTC	
GGB250	5-detct (leu2)	AAGAAGCCGTGAAACAAGTC	
GGB460	3-detect (leu2)	CACCTGGGTATTGATATGCTG	
PR00127	5-detect ura3)	TTCCTTAGTGGTATCAACGTC	
PR00128	3-detect ura3)	CAATCAAAGGTGGTCCTTCTAG	
GGB1293	5 detect dig1	CATACATACATTCACACACAC	*DIG1* deletion confirmation
GGB1294	3 detect dig1	GTGGTATTAGGAATGTCATC	
GGB1295	dig1 check fwd	GCTGATGTCGCTCTTAGAATTG	
GGB1296	dig1 check rev	TGGTGCTCTTGATCTTATTGG	
GGB1327	Dig1 orf-StuI fwd	AatcttaggcctATGTCCAGCAAAGTTGAAATTC	For construction of *pACTS-DIG1* expression plasmid
GGB1332	Dig1 orf-HindIII rev	TGAAAGCTTGTGGACTTTCTGCTCTAACC	
GGB1284	MFa-GFP fwd	TCAAACAAATAACTCTCAATTCTTATAAACTATATTAATTCAATCAACAAATTCAAATAAAAAAATATAAATAGTCGACAAAG	*MFA1p-GFP* reporter
GGB1285	MFa-GFP rev	TGCCATTGATATTGCATTTATATCCTATTGTTGAACAGTGACAGCACCTGTGGCGGTGGCTAgaccacctttgattgtaaatag	
GGB1317	MFa 5’ detect	GTAAAGAGGTGAGCGTATG	For confirmation of GFP cassette integration
PR00083	GFP detect rev	AGCATTGAAGACCATACGCG	
LT1696	CPH1 5'flank top	ACAAAACATCGAACAACCTTC	Fusion PCR for *CPH1* first copy KO
LT1697	CPH1 5'flank bottom	CACGGCGCGCCTAGCAGCGGTAATTGACATGGCGAAA	

		GAG	
LT1698	CPH1 3'flank top	GTCAGCGGCCGCATCCCTGCTTTTTCTTCCATCGTCGTC	
LT1699	CPH1 3'flank bottom	TTATATACGCAGCTTAGATGG	
LT1700	CPH1 check left	ATACTTCTGTCCACACACACAC	*CPH1* deletion confirmation
LT1701	CPH1 check right	ATCCAAACCAAACCATGTAG	
LTG01	CPH1-TAP fwd	TGATGTTGTGAATTCCAAAGTCACCAAAGTGATCAATAAAGAAGAAGTAAAACAGTCACAAACAGGTCGACGGATCCCCGGGTT	*TAP-ARG4* cassette flanked by *CPH1*
LTG02	CPH1-TAP rev	AATTTATTTGAATCCCATTTACTTAGTTTTTTTCTTTCTTTCTCTTTCTCTCTGTATCTATTCATTCGATGAATTCGAGCTCGTT	
LTG03	TAP tag check rev	ATTCTCGCTAGCAGTAGTTG	Confirm the *TAP*-tagged strain
LTG04	CPH1-TAP check fwd	TACATGAATTCCAATGGTGC	Confirm the *CPH1-TAP*-tagged strain
LT1723	TEC1 5'flank top	AATTAGACAGGGACCTTGAC	Fusion PCR for *TEC1* first copy KO
LT1724	TEC1 5'flank bottom	CACGGCGCGCCTAGCAGCGGAATAGGAATTGACAGCAACC	
LT1725	TEC1 3'flank top	GTCAGCGGCCGCATCCCTGCATCACTTACTCACTGTTGGATAC	
LT1726	TEC1 3'flank bottom	ACAAAATGCTCAATAACCTC	
LT1727	TEC1 check left	TGCACCTCAAATACAAACAAC	*TEC1* deletion confirmation
LT1728	TEC1 check right	ACGATTTGCAAGATTACACTC	
LTG05	TEC1-TAP fwd	TCACGAATAATCAAGATTATCAATTTGGTAATATAGGATACACAGAAGGATTTACTAGTGAGTTTGGTCGACGGATCCCCGGGTT	*TAP-ARG4* cassette flanked by *TEC1*
LTG06	TEC1-TAP rev	TAAACTAATGTATCCAACAGTGAGTAAGTGATAATATTTTCTTCTCTTTTCTCATTTTGTT	

		CATCGATGAATTCGAGCTC GTT	
LTG07	TEC1-TAP check fwd	AGACACCATTCATTCAGCAT C	Confirm the *TEC1-TAP*-tagged strain
LT1678	CEK1 5'flank top	ACCAACTCCAATTCCAACTC	Fusion PCR for *CEK1* first copy KO
LT1679	CEK1 5'flank bottom	CACGGCGCGCCTAGCAGC GGTTCTATTGATGACGTGGT CG	
LT1680	CEK1 3'flank top	GTCAGCGGCCGCATCCCTG CAAACTATAGGTTGGGCTT GG	
LT1681	CEK1 3'flank bottom	TTTTACGCTTGTTCAGGAAC	
LT1682	CEK1 check left	AAGGACAAGACAAGACAAG AC	*CEK1* deletion confirmation
LT1683	CEK1 check right	TATTACTCCAACTCCAACAC TC	
LTG08	CEK1-TAP fwd	AAAGATCAATTAACAATTGA AGATTTGAAAAAATTGTTAT ATGAAGAGATTATGAAACCA TTAGGTCGACGGATCCCCG GGTT	*TAP-ARG4* cassette flanked by *CEK1*
LTG09	CEK1-TAP rev	AGCCCAACCTATAGTTTTTA GTTTAGTTTAGTTTAGTTTA GTTTAGCTTAACTTRGCTTG ACCTCTTCGATGAATTCGAG CTCGTT	
LTG10	CEK1-TAP check fwd	AGGAACACCAAATATGGAA G	Confirm the *CEK1-TAP*-tagged strain
LT1687	CEK2 5'flank top	TGTCACAACTCTCGGAATTG	Fusion PCR for *CEK2* first copy KO
LT1688	CEK2 5'flank bottom	CACGGCGCGCCTAGCAGC GGAGTCACAGCTTTGGCTT TATG	
LT1689	CEK2 3'flank top	GTCAGCGGCCGCATCCCTG CTATTGGGTTCACTGTTCAA C	
LT1690	CEK2 3'flank bottom	AATGTCATTGCTCTTTGCTG	
LT1691	CEK2 check left	TGGTGACTAATTTGATCCAG	*CEK2* deletion confirmation
LT1692	CEK2 check right	AGGAACGTCTACAATGGTT G	
LTG11	CEK2-TAP fwd	ATAGATAAGAAGAATTTGGA CACCAATGACTTGAAAAAAC AAATTTTCGAAATAGTCATG	*TAP-ARG4* cassette flanked by *CEK2*

		TCGGGTCGACGGATCCCCG GGTT	
LTG12	CEK2-TAP rev	ATCTTTATAAAGTATAATAAA ATAATTTCAAACATTAGTAA AAGTAATTAATTAAGTAAAG TATCGATGAATTCGAGCTCG TT	
LTG13	CEK2-TAP check fwd	TGGCTGAACTTTTGACTTAC	Confirm the *CEK2-TAP*-tagged strain
LT2694	DIG1 5'flank top	TTACCAATCCAATCCAATCC	Fusion PCR for *DIG1* first copy KO
LT2695	DIG1 5'flank bottom	CACGGCGCGCCTAGCAGC GGTGGTTGGCGAATGAATT AAAG	
LT2696	DIG1 3'flank top	GTCAGCGGCCGCATCCCTG CCGAGATGATTAATGAGTAC ACC	
LT2697	DIG1 3'flank bottom	TTTTTCAGGCAGACCATACG	
LT2698	DIG1 check left	ATCCCACTTCACAACAACAC	*DIG1* deletion confirmation
LT2699	DIG1 check right	TCTGCTCTAACCTATCAACG	
LTG14	DIG1-TAP fwd	AATAAAAAGAAATTCTTGAA AATATGTGAAACTTGCTGGG ATCAAGTGTTTAATAAACGA GATGATGGTCGACGGATCC CCGGGTT	*TAP-ARG4* cassette flanked by *DIG1*
LTG15	DIG1-TAP rev	AAATCCTAATTAAATTTTTTT GAAAAAAAAAAAAAATAAAG ATAGAATATGATTATTGGTG TACTCATCGATGAATTCGAG CTCGTT	
LTG16	DIG1-TAP check fwd	TGATTTCCATAAAGCTGCTC	Confirm the *DIG1-TAP*-tagged strain
LT2015	GPR1-5' flank-FWD	ATAAATTAGAGGCGAGAAGC	Fusion PCR for *GPR1* KO
LT2016	GPR1-5' flank-REV	CACGGCGCGCCTAGCAGCGGT GGACAATAGACTGTAAGGAC	
LT2017	GPR1-3' flank-FWD	GTCAGCGGCCGCATCCCTGCA ACACCTTCAAATGAGGAGTC	
LT2018	GPR1-3' flank-REV	AGAGAGACACACACACAAAGT G	
LT2019	GPR1-Check-FWD	AAGGAAGGAGAGAAGAGAAC	*GPR1* deletion confirmation
LT2020	GPR1-Check-REV	TGATGTAGTGACGAAATGTG	
LT2021	GPR1-ORF-FWD	AGCACCGATGATTCTACTAC	
LT2022	GPR1-ORF-REV	ATCCACCTTCTACTCTATTG	
GGB948	HGT12 upstream fwd	TATGGGCCCCTTCAGCCTC	Fusion PCR for

		GTCTCCTTAC	*HGT12* KO
GGB949	HGT12 upstream rev	CACGGCGCGCCTAGCAGCGGCTCGAGGAGAACTTCAGATGAATGTGGG	
GGB950	HGT12 downstream fwd	GTCAGCGGCCGCATCCCTGCCCGCGGACTGGAACCTAACTATATGGC	
GGB951	HGT12 downstream rev	TACGAGCTCAGGTTATGATTACTCCAGTTGC	
GGB952	5 detect HGT12	TCGTTGAGCATCGTACCATTTC	*HGT12* deletion confirmation
GGB953	3 detect HGT12	TATCAACGTCGTGCAACTG	
GGB954	HGT12 check fwd	TGCTGTCACTGCTTCATAC	
GGB955	HGT12 check rev	GAACGCTTGAAGAATTGAG	
LT549	RT-MFA1 fwd	ATGGCTGCTCAACAACAATC	q-RT-PCR
LT550	RT-MFA1 rev	AACAGAACAAGTGGAACAGC	
LT432	RT-MFα fwd	TGACAGTAACCAAGTTGTTG	
LT433	RT-MFα rev	AGCACCAGAGGTAAGAGTAG	
LT660	RT- FIG1 fwd	AGAAGCTATGACTTGGACAGC	
LT661	RT- FIG1 rev	AGTGGTTGTTGTTGGTGTTG	
LT662	RT- FUS1 fwd	TAGCAAAAGCTCTCCAAATG	
LT663	RT- FUS1 rev	TGCGATGTAGATGGTACTTTC	
LT439	RT-STE2 fwd	tactggttggtatgatggatc	
LT440	RT-STE2 rev	aaggcaacaacaatcaatcc	
LT441	RT-STE3 fwd	tgttggtaagttggatgctg	
LT442	RT-STE3 rev	tgcatatcttgatcctgtcac	
GGB958	RT-STE4 fwd	ATGTGCCTTATGGGACTTGAC	
GGB959	RT-STE4 rev	AGCACTACCATCAGAACTTC	
GGB956	RT-CEK1 fwd	CTCAAGCTCAAGCACAACAAC	
GGB957	RT-CEK1 rev	GTGATGGTTTATGAATGGCTG	
GGB960	RT-CEK2 fwd	GTACATTTACCCACAGAGAC	
GGB961	RT-CEK2 rev	TCGAACTATATTGTCGTGG	
LWH389	RT-CPH1 fwd	ATCGACCAAACCAAGTAATG	
LWH390	RT-CPH1 rev	TCTTCAGAATTTTGAGTGGT	
LT948	RT-TEC1 fwd	AGGTACACGAATCTTCCAAG	

LT949	RT-TEC1 rev	TTGGTTGAGACTAGATGGA G	
LTG14	RT-ACT1 fwd	TAAGATTATTGCTCCACCAG	
LTG15	RT-ACT1 rev	ACCAGATTCGTCGTATTCTT G	
GGB1380	RT-WH11 fwd	CGAATCCAAATTAACTCCAG	
GGB1381	RT-WH11 rev	GAGTCACCAAAAATAGCATC	
GGB1386	RT-MET3 fwd	AACCATTAGAGCTGCTCAA G	
GGB1387	RT-MET3 rev	TCACCACCCATTCTCATAG	
GGB1388	RT-GIT3 fwd	TCCGAGTTGAGTTATGGCT G	
GGB1389	RT-GIT3 rev	AACAGATGTGACTACTTCG G	
GGB1402	RT-DLD1 fwd	GTGGAAGATTAAGCAAACG C	
GGB1403	RT-DLD1 rev	GTCTGAGGATGTTGGCAAA G	
GGB1404	RT-OP4 fwd	ACAAAGAAGAGCTGACAGT G	
GGB1405	RT-OP4 rev	TGGACAATAGCACCTTGAA C	
GGB1406	RT-WOR1 fwd	AGGTAGTATCACTCACACTG	
GGB1407	RT-WOR1 rev	ATAGCAGCTGAAACCATAG G	
GGB1410	RT-IFF6 fwd	TGTATTTCCGCTGATAGAG	
GGB1411	RT-IFF6 rev	CCTCTGACTGTGTATGTAGC	
GGB1412	RT-OFI1 fwd	TCACCTCATCAACTTCAAC	
GGB1413	RT-OFI1 rev	GAGAATATGACTGAGCTTGT G	

References:

1. Huang G, Srikantha T, Sahni N, Yi S, Soll DR. CO(2) regulates white-to-opaque switching in Candida albicans. *Curr Biol* **19**, 330-334 (2009).

2. Noble SM, French S, Kohn LA, Chen V, Johnson AD. Systematic screens of a Candida albicans homozygous deletion library decouple morphogenetic switching and pathogenicity. *Nature genetics* **42**, 590-598 (2010).

3. Huang G, Wang H, Chou S, Nie X, Chen J, Liu H. Bistable expression of WOR1, a master regulator of white-opaque switching in Candida albicans. *Proc Natl Acad Sci U S A* **103**, 12813-12818 (2006).

4. Noble SM, Johnson AD. Strains and strategies for large-scale gene deletion studies of the diploid human fungal pathogen Candida albicans. *Eukaryot Cell* **4**, 298-309 (2005).

5. Tao L*, et al.* White cells facilitate opposite- and same-sex mating of opaque cells in Candida albicans. *PLoS Genet* **10**, e1004737 (2014).

6. Scaduto CM*, et al.* Epigenetic control of pheromone MAPK signaling determines sexual fecundity in Candida albicans. *Proc Natl Acad Sci U S A* **114**, 13780-13785 (2017).

7. Chen J, Lane S, Liu H. A conserved mitogen-activated protein kinase pathway is required for mating in Candida albicans. *Mol Microbiol* **46**, 1335-1344 (2002).

8. Liang W, Guan G, Li C, Nobile CJ, Tao L, Huang G. Genetic regulation of the development of mating projections in Candida albicans. *Emerg Microbes Infect* **9**, 413-426 (2020).

9. Du H*, et al.* The transcription factor Flo8 mediates CO2 sensing in the human fungal pathogen Candida albicans. *Mol Biol Cell* **23**, 2692-2701 (2012).

10. Guan G*, et al.* Environment-induced same-sex mating in the yeast Candida albicans through the Hsf1-Hsp90 pathway. *PLoS Biol* **17**, e2006966 (2019).
