# Supplementary material for: Glucose depletion enables Candida albicans mating independently of the epigenetic white-opaque switch
Source: Nat Commun. 2023 Apr 12;14:2067. doi: 10.1038/s41467-023-37755-8 (PMC10097730; doi:10.1038/s41467-023-37755-8)
Supplement: Supplementary file 2 — Description of Additional Supplementary Files [file 41467_2023_37755_MOESM2_ESM.pdf]

Description of Additional Supplementary Files:

Supplementary Dataset 1: Protein expression profiles of the MTLa wor1Δ/Δ cells grown on YP-K and YPD-K media. Protein IDs were according to the Uniprot database. Gene names and descriptions were according to the CGD database (http://www.candidagenome.org/). wor1Δ/Δa YP-K vs wor1Δ/Δa YPD-K ratio=Log2 Ratio(abundance value of wor1Δ/Δa YP-K/abundance value of wor1Δ/Δa YPD-K). Genes were classified according to Lan, et al., PNAS, 2002). This Dataset is associated with Figure S2.
