# Supplementary material for: Glucose depletion enables Candida albicans mating independently of the epigenetic white-opaque switch
Source: Nat Commun. 2023 Apr 12;14:2067. doi: 10.1038/s41467-023-37755-8 (PMC10097730; doi:10.1038/s41467-023-37755-8)
Supplement: Supplementary file 4 — Reporting Summary [file 41467_2023_37755_MOESM4_ESM.pdf]

nature portfolio

Corresponding author(s): Guanghua Huang

Last updated by author(s): Mar 30, 2023

Reporting Summary

Nature Portfolio wishes to improve the reproducibility of the work that we publish. This form provides structure for consistency and transparency in reporting. For further information on Nature Portfolio policies, see our Editorial Policies and the Editorial Policy Checklist.

Statistics

For all statistical analyses, confirm that the following items are present in the figure legend, table legend, main text, or Methods section.

n/a	Confirmed	
☐	☒	The exact sample size (*n*) for each experimental group/condition, given as a discrete number and unit of measurement
☐	☒	A statement on whether measurements were taken from distinct samples or whether the same sample was measured repeatedly
☐	☒	The statistical test(s) used AND whether they are one- or two-sided<br>*Only common tests should be described solely by name; describe more complex techniques in the Methods section.*
☒	☐	A description of all covariates tested
☒	☐	A description of any assumptions or corrections, such as tests of normality and adjustment for multiple comparisons
☐	☒	A full description of the statistical parameters including central tendency (e.g. means) or other basic estimates (e.g. regression coefficient) AND variation (e.g. standard deviation) or associated estimates of uncertainty (e.g. confidence intervals)
☐	☒	For null hypothesis testing, the test statistic (e.g. *F*, *t*, *r*) with confidence intervals, effect sizes, degrees of freedom and *P* value noted<br>*Give P values as exact values whenever suitable.*
☒	☐	For Bayesian analysis, information on the choice of priors and Markov chain Monte Carlo settings
☒	☐	For hierarchical and complex designs, identification of the appropriate level for tests and full reporting of outcomes
☒	☐	Estimates of effect sizes (e.g. Cohen's *d*, Pearson's *r*), indicating how they were calculated

Our web collection on statistics for biologists contains articles on many of the points above.

Software and code

Policy information about availability of computer code

Data collection	Bio-Rad CFX Manager3.1 for Bio-Rad Real-Time PCR System.
Data analysis	The raw Proteomic data were processed using Proteome Discoverer software (version 2.4, Thermo Fisher Scientific) with in-house Mascot search engine (version 2.7.0, Matrix Science).<br>Differentially enriched or expressed proteins in quantitative mass spectrometry-based proteomics data were tested using the DEP package version 3.16.<br>Western blotting data were analyzed using Image Lab 3.0.<br>Statistical data were analyzed using Graphpad Prism 9.0.

For manuscripts utilizing custom algorithms or software that are central to the research but not yet described in published literature, software must be made available to editors and reviewers. We strongly encourage code deposition in a community repository (e.g. GitHub). See the Nature Portfolio guidelines for submitting code & software for further information.

Data

Policy information about availability of data

All manuscripts must include a data availability statement. This statement should provide the following information, where applicable:

- Accession codes, unique identifiers, or web links for publicly available datasets
- A description of any restrictions on data availability
- For clinical datasets or third party data, please ensure that the statement adheres to our policy

The authors declare that the data supporting the findings of this study are available within the article and its Supplementary Information files. The mass spectrometry proteomics data have been deposited to the ProteomeXchange Consortium (http://proteomecentral.proteomexchange.org) via the iProX partner repository with the dataset identifier PXD040559. Source data are provided in this paper.

Human research participants

Policy information about studies involving human research participants and Sex and Gender in Research.

Reporting on sex and gender	not applicable
Population characteristics	not applicable
Recruitment	not applicable
Ethics oversight	not applicable

Note that full information on the approval of the study protocol must also be provided in the manuscript.

Field-specific reporting

Please select the one below that is the best fit for your research. If you are not sure, read the appropriate sections before making your selection.

☒ Life sciences ☐ Behavioural & social sciences ☐ Ecological, evolutionary & environmental sciences

For a reference copy of the document with all sections, see nature.com/documents/nr-reporting-summary-flat.pdf

Life sciences study design

All studies must disclose on these points even when the disclosure is negative.

Sample size	No statistical methods were used to predetermine sample size. Sample size was determined based on standards of the field and the preliminary experiments. 3 - 5 sample size was used for analysis in this study. These sample sizes were sufficient to investigate meaningful biological difference with good reproducibility.
Data exclusions	No data was excluded from the analysis.
Replication	All experiments were performed at least two times independently and successfully reproduced. The number of replicates is indicated in the corresponding figure legend and/or in the corresponding material and method section.
Randomization	Randomization is not relevant for this study. Candida albicans cells were cultured, harvested, processed under identical and standard conditions. Microscopic images were randomly taken and selected.
Blinding	Blinding was not relevant for this study, as all experiments were in vitro and no subjective data analysis was involved. qPCR and mating assys were performed with samples just numbered as 1, 2, 3 etc. and experimenters were not aware of the identity of the samples.

Reporting for specific materials, systems and methods

We require information from authors about some types of materials, experimental systems and methods used in many studies. Here, indicate whether each material, system or method listed is relevant to your study. If you are not sure if a list item applies to your research, read the appropriate section before selecting a response.

Materials & experimental systems

n/a	Involved in the study	
☐	☒	Antibodies
☒	☐	Eukaryotic cell lines
☒	☐	Palaeontology and archaeology
☒	☐	Animals and other organisms
☒	☐	Clinical data
☒	☐	Dual use research of concern

Methods

n/a	Involved in the study	
☒	☐	ChIP-seq
☒	☐	Flow cytometry
☒	☐	MRI-based neuroimaging

Antibodies

Antibodies used	For western blotting, horseradish peroxidase (HRP)-conjugated monoclonal anti-TAP antibody (1:1,000 dilution, clone number (not available), catalog number P1291, Sigma-Aldrich, Inc), commercial mouse monoclonal anti-Cdc28 antibody (1:1000 dilution, clone number G-7; catalog number sc-515762, Santa Cruz Biotechnology, Inc), anti-mouse IgG, HRP-linked antibody (1:2000 dilution, clone number (not available), catalog number 7076S, Cell Signaling Technology Inc) were used.
Validation	All the antibodies were commercial antibodies with quality control and validation statements provided on the manufacturer's website:<br>horseradish peroxidase (HRP)-conjugated monoclonal anti-TAP antibody (1:1,000 dilution, P1291, Sigma-Aldrich, Inc), (https://www.sigmaaldrich.cn/CN/zh/product/sigma/p1291. mouse monoclonal anti-Cdc28 antibody (1:1000 dilution, sc-515762, Santa Cruz Biotechnology, Inc), (https://www.scbt.com/p/cdc28-antibody-g-7?requestFrom=search). anti-mouse IgG, HRP-linked antibody (1:2000 dilution, 7076S, Cell Signaling Technology Inc), (https://www.cellsignal.com/products/secondary-antibodies/anti-mouse-igg-hrp-linked-antibody/7076).
